# Gene expression analysis of rocket salad under pre-harvest and postharvest stresses: A transcriptomic resource for *Diplotaxis tenuifolia*

**DOI:** 10.1371/journal.pone.0178119

**Published:** 2017-05-30

**Authors:** Marina Cavaiuolo, Giacomo Cocetta, Natasha Damiana Spadafora, Carsten T. Müller, Hilary J. Rogers, Antonio Ferrante

**Affiliations:** 1 Department of Agricultural and Environmental Sciences, Università degli Studi di Milano, Milano, Italy; 2 School of Biosciences, Cardiff University, Cardiff, United Kingdom; Institute of Genetics and Developmental Biology Chinese Academy of Sciences, CHINA

## Abstract

*Diplotaxis tenuifolia* L. is of important economic value in the fresh-cut industry for its nutraceutical and sensorial properties. However, information on the molecular mechanisms conferring tolerance of harvested leaves to pre- and postharvest stresses during processing and shelf-life have never been investigated. Here, we provide the first transcriptomic resource of rocket by *de novo* RNA sequencing assembly, functional annotation and stress-induced expression analysis of 33874 transcripts. Transcriptomic changes in leaves subjected to commercially-relevant pre-harvest (salinity, heat and nitrogen starvation) and postharvest stresses (cold, dehydration, dark, wounding) known to affect quality and shelf-life were analysed 24h after stress treatment, a timing relevant to subsequent processing of salad leaves. Transcription factors and genes involved in plant growth regulator signaling, autophagy, senescence and glucosinolate metabolism were the most affected by the stresses. Hundreds of genes with unknown function but uniquely expressed under stress were identified, providing candidates to investigate stress responses in rocket. Dehydration and wounding had the greatest effect on the transcriptome and different stresses elicited changes in the expression of genes related to overlapping groups of hormones. These data will allow development of approaches targeted at improving stress tolerance, quality and shelf-life of rocket with direct applications in the fresh-cut industries.

## Introduction

Plants cope with environmental stresses by coordinately activating and repressing large numbers of genes that modulate their physiology and metabolism to enable survival [1]. In a primary stress response plants immediately activate a set of genes during the first few hours [2]. Their expression is mainly transient and they regulate a class of secondary genes that are associated with defence mechanisms and longer-term responses [3,4].

Gene expression varies temporally and in specificity to the stress imposed. In *Arabidopsis* ~30% of the transcriptome is induced by stresses and the majority of changes in gene regulation are stimulus-specific, increasing in specificity with the time of exposure to the stress [5].

Plant growth regulators (PGRs), abscisic acid (ABA), ethylene (ET), jasmonate (JA) and salicylic acid (SA) are key mediators of stress responses [6], although auxin (AUX), gibberellic acids (GAs) and brassinosteroids (BRs) are also involved in a complex interplay that regulates down-stream responses [7]. Dehydration or salinity activate both ABA-dependent and independent pathways which converge in the activation of stress-responsive transcription factors (TFs) [8]. Wounding and drought induce a transient induction of JA biosynthesis [9]. The role of auxin is less clear but there appears to be crosstalk at the level of TFs, which are activated both by ABA and ethylene [6]. A crosstalk between JA and AUX-IAA signaling pathways also occurs at metabolic-level: indole-3-acetic acid (IAA) amidohydrolase (IAH) genes are activated by wounding and coordinate stress responses by promoting IAA signaling and attenuating JA signaling [10]. Most ethylene biosynthesis and response genes are up-regulated rapidly but transiently [2,11].

Stress-dependent regulation of GA signaling is involved in modulating reactive oxygen species (ROS) to promote stress tolerance [12]. A protective role for BRs in resistance against drought and cold was shown in *Arabidopsis* and *Brassica napus* during early time points of stress [13].

TFs dominate the rapid response genes: many of them regulate PGR biosynthesis and signaling, e.g. the C_2_H_2_ Zinc finger proteins *AZF2*, *ZAT10* and *ZAT1* [2] involved in JA-early signaling response. Other important TFs are those involved in ethylene and ABA signaling pathways. The *ABA RESPONSE ELEMENT* (*ABRE*) *BINDING FACTORS (AREB/ABF)* TFs bind to *ABRE cis*-elements in the promoters of downstream genes and regulate osmotic stress tolerance in an ABA-dependent manner [14].

Abiotic stresses induce premature leaf senescence inducing yield losses in crops [15]. Senescence-associated TFs are expressed before senescence symptoms appear, e.g. the *WRKY* and *NAC* gene family [16,17], and many downstream genes are activated to counteract cell damage or delay the onset of senescence. An early response to stress is a rise in ROS [4], which induces increased activity of antioxidant enzymes, including superoxide dismutase (SOD) and catalase (CAT), that ensure the maintenance of ROS homeostasis [18]. The ascorbate-glutathione pathway and tocopherols also buffer the cell against ROS [19].

Autophagy removes macromolecules damaged during stress [20] and autophagy-related genes are up-regulated within 2–4 h after stress induction by drought or salinity [21]. Autophagy is regulated at least partly via ABA [22].

Glucosinolate (GLS) content increases markedly under salinity, drought, high temperature and nitrogen (N) deficiency, and several *MYB* TFs are involved in GLS accumulation [23].

In leafy crops such as salads, pre-harvest and postharvest stresses, even of short duration, contribute to changes in metabolism that accelerate quality losses [24,25]. Hence, leafy vegetables are preferably processed within 24-48h postharvest. Pre-harvest stresses include nutrient status, particularly N-starvation, excessive salinity and heat just prior to harvest [26]. N-deficiency is often imposed in hydroponically grown leafy salads such as rocket to avoid excess nitrate content at harvest. Excess salinity can also be a problem in hydroponic systems, especially those that use high electrical conductivity (EC) water. In soil-grown systems high temperatures (up to 40°C) during the harvest period are also not uncommon, especially in greenhouse grown crops. Postharvest, leaves are typically stored and transported at 0–5°C to delay deterioration, thus imposing potential cold stress [27]. However, temperatures up to 20°C can be reached if the leaves are transported in bulk and not sufficiently cooled during harvest, subjecting them to dehydration stress. Processing also results in mechanical damage.

To date there is a lack of studies focusing on the effects of pre- and postharvest stresses on gene expression in leafy crops. Understanding these effects on fresh-cut vegetables is important to reduce the rapid deterioration and short shelf-life of these products as well as to ensure their nutritional value.

Two *Brassicaceae* species, known as rocket salad (*Eruca sativa* and *Diplotaxis tenuifolia*) are of increasing commercial importance and constitute a good source of anti- carcinogens [28]. As for many horticultural crops, transcriptomic resources for rocket are lacking. Genetic resources would be of substantial benefit in breeding for improved stress-resilience, water efficiency and better postharvest storage, as has been found for other crops [29].

We used RNA-Seq to generate the first transcriptome of rocket (*Diplotaxis tenuifolia*) leaves and study gene expression following commercially relevant abiotic stress treatments pre-harvest and postharvest. Stresses were imposed for 24h to investigate early secondary responses, where stress responses have been activated but cellular functions are still unimpaired [30,31].

## Materials and methods

### Plant materials and stress treatments

*Diplotaxis tenuifolia* cv Frastagliata plants were grown hydroponically in controlled growth chambers (26°C; 400 Wm^-2^, 16 h photoperiod; relative humidity 60–70%) for 30 days post-sowing. Seeds were sown in polystyrene trays within tanks using perlite as growth substrate. Tanks were filled with nutrient solution composed of (concentrations expressed in mM): 13 N-NO_3_, 1.5 P, 8 K, 3.5 Ca, 1.7 Mg, 9.5 Na, 8.0 Cl, 2.7 S, 0.04 Fe and micronutrients (Hoagland's solution). Stresses were applied for 24h after 30 days growth. Pre-harvest stresses were imposed before harvesting by transferring plants to fresh nutrient solution as follows: (1) salinity: containing 250 mM NaCl; (2) root-imposed heat stress: heating to 40°C in a water bath; (3) N-starvation: devoid of nitrogen (-NO_3_^-^/-NH_4_^+^). Postharvest stresses were applied to freshly harvested leaves as follows: (1) cold stress: leaves were stored in closed plastic boxes in the dark at 4°C; (2) wounding: leaves were cut into 4–6 pieces and stored in sealed plastic boxes in the dark at 20°C; (3) dehydration: leaves were stored in open plastic boxes in the dark at 20°C; (4) dark: leaves were sealed in plastic boxes in the dark at 20°C. Control leaves for pre- and postharvest stress treatments were collected from untreated plants to account for any circadian rhythms and ensure that they were completely unstressed.

### RNA-Seq and bioinformatic analysis

Total RNA was extracted from 1 g of leaf powder using the hot borate method [32]. RNA purity and integrity were assessed with an Agilent 2100 bioanalyzer-RNA 6000 Nano Chip (Agilent Technologies) and Nanodrop 8000 (Thermo Scientific). RNA with A260/A280 ≥ 1.8 and RNA integrity number (RIN) ≥ 6 was used for RNA-Seq. RNA integrity was also checked on ethidium bromide stained agarose gels. A single-replicate RNA-Seq experiment for each sample was generated. Library preparation, Illumina sequencing and *de novo* assembly were performed at the Institute of Applied Genomics (IGA) (Udine, Italy). Random primed cDNA libraries were prepared with a TruSeq RNA Sample Prep kit (Illumina) and sequenced in paired-end mode in one run on the Illumina HiSeq™-2000 Platform, generating up to 30 M reads of 100 bp per sample. Sequences derived from contaminating microorganisms were removed from the raw data using ERNE-FILTER. Adaptor trimming, quality control and *de novo* assembly were performed using CLC Genomics Workbench (http://www.clcbio.com). Adapters were trimmed using mismatch costs of 2 and a gap (insertion or deletion) costs of 3, internal matches with a minimum score of 10 and end matches with a minimum score of 4. Quality control was applied by: (1) trimming based on quality scores set to 0.05; (2) trimming of ambiguous nucleotides/undetermined nucleotides (N) with a maximum of 2 ambiguous nucleotides allowed. The quality score based trimming used a modified-Mott trimming algorithm, where the quality score (Q) per base was converted to an error probability according to *P*_error_ = 10^{Q/-10}^ where *P* is the base-calling error probability. The *P*_*error*_ was subtracted from the 0.05 limit for each base and the cumulative total of the values calculated for each read. If the sum was negative, it was set to zero and the read sequence starting at the first positive value and ending at the highest value of the cumulative total was retained. To trim ambiguous nucleotides, the algorithm found the maximum length region containing < 2 ambiguities and trimmed the ends not included in this region. Processed reads from the eight libraries were pooled (total 3 x 10^8^ sequences) and *de novo* assembled into 33974 contigs. The CLC *de novo* assembly uses the de Bruijn graphs algorithm to break reads into smaller sequences of DNA (k-mers or word size). D*e novo* assembly used a word size of 20; a bubble size of 50; a minimum contig length of 400-bp: scaffolding; 2 mismatches allowed and insertion and deletion costs set to 3. Sequences spanned 45166539 bp with an average length of 1333 bp. Two approaches were used to check assembly quality: 1) aligning the assembled contigs to the *Arabidopsis* genome (since *Arabidopsis* is a closely related species); 2) paired-end reads were mapped back to the assembled contigs. For back-mapping, identical mismatch, insertion and deletion costs were used as for the *de novo* assembly but with a word size of 61; bubble size of 5000, minimum contig length of 400-bp, the length fraction set to 0.9 and similarity to 0.95. Thus, at least 90% of the individual reads needed to have at least 95% identity with the contig sequence to be included in the mapping. 18655126 single reads and 25606010 paired reads were not mapped. The sequencing data were deposited in the NCBI Sequence Read Archive (http://www.ncbi.nlm.nih.gov/Traces/sra) under SRP study accession number SRP102718.

### Annotation and gene expression analysis

BLAST alignments with a cut-off E-value of 10^−3^, GO term mapping, RPS-BLAST to enzymes and Pfam domains were obtained with FastAnnotator [33]. BLAST redundancy was determined by counting the number of unique hits (a single contig matching a single protein) and redundant hits (multiple contigs matching the same protein). GO enrichment analysis was run using the "Plant_GOslim" ancestor terms from CateGorizer by multiple count [34]. KAAS web-based software was used to query the Kyoto Encyclopedia of Genes and Genomes (KEGG) [35] database using *Arabidopsis* and the bidirectional best hit assignment method (BBH) to automatically generate KEGG pathways.

RPKMs [36] were computed from the raw counts to rank the expressed genes in each single dataset. A threshold of RPKM ≥ 3 was set to define transcriptionally active genes within each library [37]. Statistical analysis for differential expression between control and treated samples was performed with the R package EdgeR using the trimmed mean of M-values (TMM) as normalization method [38,39], shown to be preferable to RPKM values [40]. A matrix of read counts for each contig in the treated libraries and control was created and used as input for edgeR.

Knowing that the absence of biological replicates implies limitations in testing for differential expression, analysis included three components. (1) a requirement for expression of the transcripts by setting a threshold of 10 mapped reads per transcript in the dataset being compared (stress *vs*. control) regardless of the corresponding RPKM value. This allowed removal of genes with very low counts that provide little statistical relevance for differential expression and influence the performance of the multiple testing adjustment while retaining condition-specific expression patterns (e.g. genes having RPKM ≥ 3 in one treatment and RPKM < 3 in another). TMM normalization was computed by taking as library sizes the column sums of the counts for each dataset. (2) a reasonable estimation of biological variation across all genes was included. A nominal average dispersion value was set to 0.25 and a Biological coefficient of variation (BCV) was set to 50% based on previous data [39,41]. This assumes the presence of variations in gene counts between different RNA samples and a relatively high coefficient of biological variation. This was used for the exactTest function according to the following script: bcv <- 0.25 y <- DGEList(counts = counts, group = group) et <- exactTest(y, dispersion = bcv^2). (3) DEGs were isolated based on FDR ≤ 0.05 and log_2_ fold-change (FC) ratio ≥ 2 or ≤ -2 between the control and each treated sample. Heatmaps were generated from TMM normalized counts per million (CPM) for each transcript in all the samples. The MA plot was generated by using “plotSmear” from the edgeR package. MDS plots, were performed to measure the relationship between samples based on multidimensional scaling through the “plotMDS.dge” function of the edgeR package.

### RT-PCR

DEGs for RT-PCR were selected based on clear up-or down-regulation in the RNA-Seq experiment. For RT-PCR, the RNA was extracted from rocket salad grown as described above using an RNA Easy kit (Qiagen). Genomic DNA was removed using DNAse (Promega) and removal was verified by PCR with Elongation Factor 1 alpha (EF1α) gene primers. cDNA was synthesized using MMLV reverse transcriptase (Promega). RT-PCR was run on an Opticon Monitor 3.1.32 (Bio-Rad Laboratories, Inc.). Primer efficiency (E) was determined by measuring the Ct of a serial dilution of cDNA according to the formula: E = (10^−1/slope^−1)×100. All PCRs displayed efficiencies between 95% and 100%. EF1α produced reproducible results across the cDNAs and was used for normalization [42,43]. PCR was performed in 20 μL reactions consisting of absolute Blue qPCR SYBR Green Mix (2X) (Thermo Scientific), dATP-dCTP-dGTP (0.4 μM) and dUTP (0.8 μM), 0.4 μM of primers (S1 Table) and cDNA (60 ng). Reactions were run in triplicate from two biological replicates and included negative controls. Cycling parameters included an initial deactivation of the Taq enzyme at 95°C for 15min, 40 cycles of denaturation (95°C for 15s), annealing and extension (60°C for 30s), followed by a melting curve (0.5°C increase every 10s from 60°C to 98°C) [44]. A unique ‘melting peak’ was obtained in every reaction. Results were analyzed using the delta-delta Ct (∆∆Ct) method [45].

## Results

### Sequencing and annotation of the rocket leaf transcriptome

**Fig 1:**
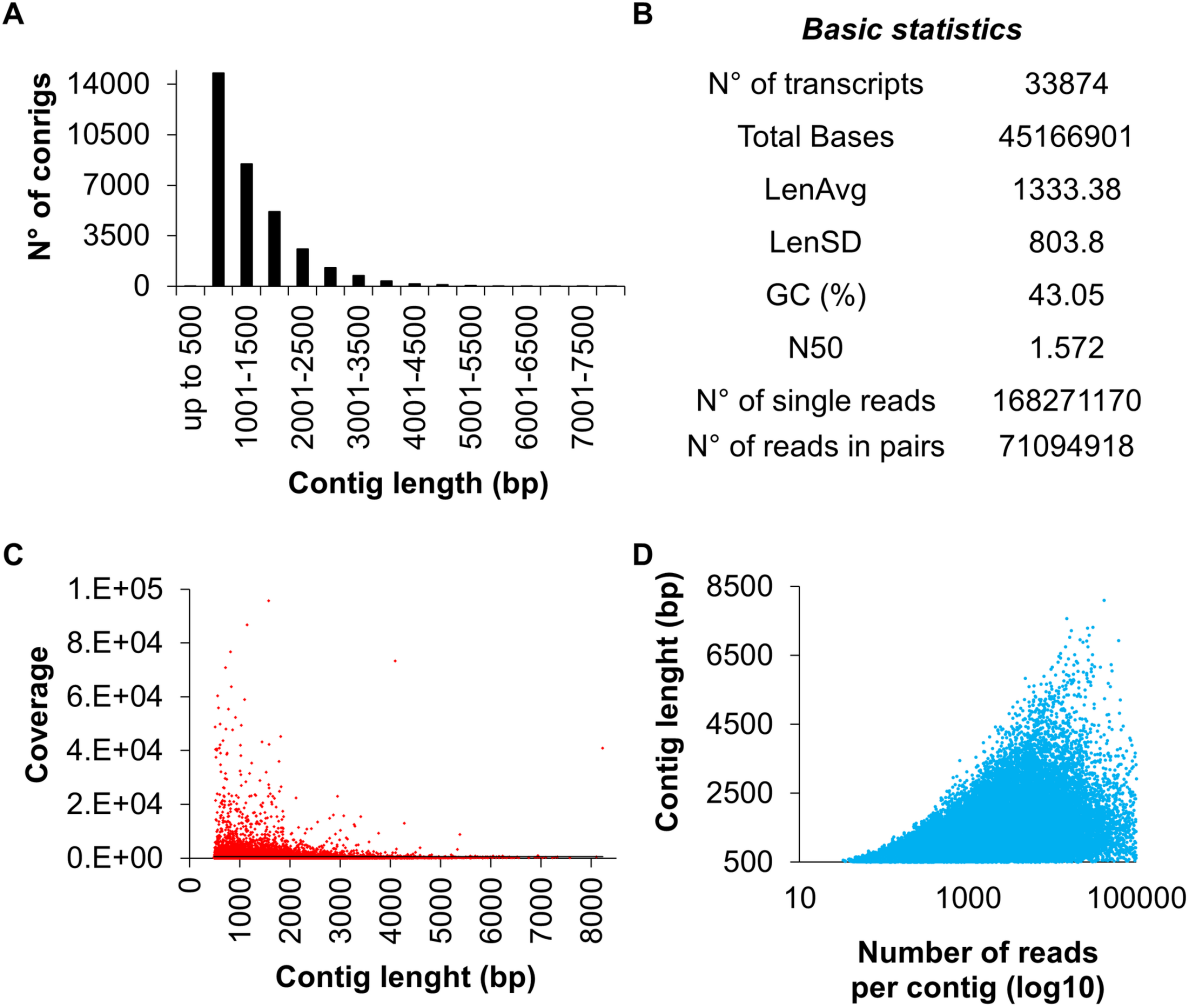
Quality of transcriptome *de novo* assembly. A) Histograms showing the assembled contigs in relation to their length. B) Statistics of the assembly. C) Contig length as a function of the contig read depth. D) Contig read number as a function of the contig length. Sequences between 500–2000 bp had the highest coverage with an average depth of 534 reads per nucleotide. Transcripts assembled with up to 5000 reads showed a linear relationship between read number and contig length. Abbreviations: Avg., average; Len., length; SD, standard deviation; N50, median weighted lengths; GC., guanine cytosine.

A transcriptome comprising 33874 contigs/transcripts (S1 Text) ranging from 486-to 8225 bp (S2 Table) was generated from *de novo* assembly of RNA-Seq data produced from leaves of baby-leaf commercial stage rocket plants subjected to seven different stresses for 24h and from a control. Pre-harvest stresses comprised nutrient solution through which 250 mM NaCl (salinity), 40°C (heat) or N-starvation stresses were imposed. Postharvest stresses were: dark storage at 4°C (cold), chopping and dark storage at 20°C (wounding), dark storage at 20°C in sealed boxes (dark) and dark storage at 20°C in open boxes (dehydration). In total, RNA-Seq generated 300 million paired-end reads of which ~99% were of high quality (Q20 bases and GC-content of ~43%) (Table 1). Over 43% of the contigs were between 500–1000 bp with a N50 value of 1.572 bases similar to that found in *A*. *thaliana* [46] and an average depth of 534 reads per nucleotide (Fig 1).

**Table 1:** Total read counts from RNA-Seq.

*Treatment*	*Raw reads*	*High quality reads*	*Unique mapping reads*	*Multi mapping reads*
Control	39883760	39533065	5080161	5080320
Cold	35877220	35557144	3801334	3801431
Dark	17778034	17548885	719523	719548
Dehydration	65444728	64931449	8877831	8878118
Wounding	49961776	49575690	7813616	7813838
Salinity	30565824	30286734	11350213	11350555
Heat	37006048	36677071	4877145	4877259
N-starvation	11915484	11839582	2484715	2484810

The numbers of raw reads and high quality reads after filtering are shown for each library. The sum of unique mapping reads and multi mapping reads is the total number of reads that are remapped to the assembled contigs.

BLAST hits were found for 30819 contigs (91%) including 21472 unigenes (Table 2; S3A Table): most of them showed 100% positive alignment and similarity to *Arabidopsis thaliana* and *Arabidopsis lyrata* sequences followed by other members of the *Brassicaceae* (S1A–S1C Fig). The redundancy level was 45.4% suggesting extensive splice variants or paralogues, while 45.6% (15459 contigs) had unique hits. 18871 contigs (61.2%) matched known proteins and 12048 (39.1%) matched proteins with unknown functions. 9% of the contigs were not annotated and may correspond to untranslated regions, pseudogenes, non-coding RNAs or genes not yet described.

**Table 2:** Functional annotation statistics of the RNA-Seq.

*BLAST hit to nr database*	*Total N° of transcripts*	*Annotation (GO*, *Domain*, *Enzyme)*
Known protein	18871	17518
Unnamed protein	1446	1350
Hypothetical protein	7365	6248
Uncharacterized protein	1705	1099
Unknown protein	460	358
Predicted protein	1072	981
No hit	3055	149
Total	33874	27703

The number of BLAST hits including known and unknown protein function (unnamed, hypothetical, uncharacterized, predicted) is reported along with the number of sequences that contain the annotation in one or all of the three functional categories: Gene Ontology (GO), Enzyme and Domain. 27703 contigs (81.7%) were completely annotated by FastAnnotator.

27703 transcripts were assigned to one or more Gene Ontology (GO) terms (S3B Table; S2 Fig). The most abundant class under “biological process” was “regulation of transcription DNA-dependent” while “ATP binding” and “plasma membrane” were the predominant classes under “molecular function” and “cellular component” respectively (S3 Fig). In the absence of a BLAST or GO match, gene functions were inferred from conserved domains and/or enzymatic activity. Multi-domain and single-domain predicted proteins were classified into Pfam families (Table 3). Leucine-rich repeat (LRR) and Pentatricopeptide repeat (PPR) were the prevalent domains followed by RNA recognition motif (RRM) and ATPases Associated with diverse cellular Activities (AAA). The most prevalent domain among TFs was the Myb_DNA-binding domain.

**Table 3:** Clustering of domain profiles and abundance of most represented Pfam domains.

*Domain*	*N° of transcripts*	*Domain*	*N° of transcripts*
PPR	6185	zf-RVT	149
LRR	3483	ABC_tran	141
RRM	1908	p450	140
AAA	1198	Kelch_1	139
Ank	1034	PHD	135
Pkinase	1033	DnaJ	131
Pkinase_Tyr	1002	PP2C	121
WD40	788	DEAD	119
Efhand	520	Arf	117
Tropomyosin	515	HLH	117
GST	512	Ubiquitin	117
FDF	500	OmpH	115
zf-RING_2	473	C2	113
zf-C3HC4	385	MatE	108
Myb_DNA-binding	378	Abhydrolase_1	104
DUF4200	287	TPT	99
ERM	273	Peptidase_C1	91
F-box	262	Thioredoxin	91
Mito_carr	253	Homeobox	88
Trichoplein	237	adh_short	86
RVT	190	Sugar_tr	83
Filament	175	bZIP_1	79
Helicase_C	155	HSP	75
Arm	153		

Enzyme commission numbers (EC) were prevalently assigned to oxidoreductases, transferases, kinases, hydrolases, isomerases and synthetases. Mapping to Kegg Orthology (KO) terms and reference canonical pathways (KEGG) (S3C and S3D Table) associated 7151 transcripts to 6647 KO terms and 313 pathways, respectively. Metabolism (741 sequences), biosynthesis of secondary metabolites (339 sequences), microbial metabolism in diverse environments (125 sequences), ribosome (118 sequences) and spliceosome (100 sequences) were the most represented pathways. BRITE object mapping (S3E Table) showed enrichment of kinases, TFs, spliceosome and ribosome related proteins, transporters and secretion system proteins.

### Differential gene expression analysis under stress

Across all treatments there were nearly 20000 transcriptionally active (Reads per kilobase per million mapped reads—RPKM ≥ 3) and around 14000 transcriptionally silent (RPKM < 3) genes (S2B Table). The number of expressed genes varied among libraries from 18131 (N-starvation) to 20879 (salinity) (Table 4). Differential gene expression (DEG) analysis (Tables 5 and 6; S4A–S4G Table) indicated that dehydration and wounding had the largest number of DEGs; in contrast, pre-harvest stresses elicited very limited DEGs (Table 5; S4A–S4G Table).

**Table 4:** Transcriptionally active genes.

*Treatment*	*Active genes*	*Silent genes*
Control	20770	13104
Cold	19301	14573
Dark	18748	15126
Dehydration	20529	13345
Wounding	20140	13734
Salinity	20879	12995
Heat	20486	13388
N-starvation	18131	15743

The number of transcriptionally active and silent genes was based on a RPKM threshold ≥ 3 and < 3 respectively.

**Table 5:** Total number of DEGs at FDR ≤ 0.05 in each library according to FC ≥ 2 and FC ≤ -2 for up-regulated and down-regulated genes respectively.

Treatment	Up-regulated genes	Down-regulated genes	No hit or annotation	Unknown
Cold	887	652	126	587
Dark	708	964	140	573
Dehydration	1372	2280	226	1305
Wounding	1444	2247	196	1315
Salinity	407	486	65	340
Heat	35	37	7	47
N-starvation	9	5	4	3

The number of genes without an annotation and resulting in unknown protein functions from a BLAST search is reported for each stress.

**Table 6:** Gene identities of the top differentially regulated genes under each stress with FC ≥ 4 and FC ≤-4.

***Contig (c)***	***FC***	***Stress***	***Putative protein***	***Function/Molecular process***
**c_6101**	7.9	D	branched-chain-amino-acid aminotransferase 2	Amino-acid biosynthesis
**c_14246**	7.4	C	C-regulated protein	Response to stress
**c_1631**	7.3	S	lipid transfer protein 4-like	Signaling. cell wall loosening
**c_2210**	7.1	S	protease inhibitor/seed storage/lipid transfer protein	Proteolysis
**c_2833**	7	DH	bifunctional lysine-ketoglutarate (LKR/SDH)	L-lysine catabolic process
**c_15577**	6.8	DH	transmembrane amino acid transporter-like protein	Amino-acid transport
**c_11470**	6.5	C	sulfotransferase family protein	Response to jasmonate
**c_9848**	6.5	C	low-temperature regulated protein BN115	Response to stress
**c_10453**	6.4	W	glutamate dehydrogenase	Amino-acid metabolism
**c_4022**	6.4	C	methylcrotonoyl-CoA carboxylase beta chain	Leucine catabolic process
**c_21786**	6.4	W	inositol oxygenase 4	Ascorbate biosynthesis
**c_3112**	6.3	DH	calcium-binding protein CML19	Ca2+ signaling
**c_394**	6.3	S	water-soluble chlorophyll protein	Endopeptidase inhibition
**c_8403**	6.2	C	ANAC019	Gene regulation
**c_15152**	5.8	D	UDP-D-glucose/UDP-D-galactose 4-epimerase 1	Sugar metabolic process
**c_3711**	5.7	W	arginine vasopressin (AVP1-2)	Proton transport
**c_22888**	5.7	W	wound-responsive 3	Transport
**c_18235**	5.7	D	methylcrotonoyl-CoA carboxylase subunit alpha	Leucine catabolic process
**c_31444**	4.1	N-st	hypothetical protein	-
**c_4295**	-4.6	H	Cu/Zn-superoxide dismutase copper chaperone precursor	Oxidation-reduction
**c_19381**	-4.8	S	germin-like protein	Disease resistance
**c_6302**	-5	C	peptide methionine sulfoxide reductase B9	Oxidation-reduction
**c_23069**	-5.1	S	invertase/pectin methylesterase inhibitor	Neg. regulation of enzyme
**c_907**	-5.3	C	subtilisin-like protease	Proteolysis
**c_2067**	-5.5	D	phosphoglycerate kinase 1	Glycolysis
**c_150**	-5.6	D	glyceraldehyde 3-phosphate dehydrogenase A subunit 2	Glucose metabolic process
**c_4002**	-5.8	W	nucleosome assembly protein 1;2	DNA repair
**c_2432**	-5.8	W	thylakoid lumenal 29.8 kDa protein	Glucosinolate metabolic process
**c_544**	-6.1	DH	glucose-1-phosphate adenylyltransferase large subunit 1	Starch biosynthesis
**c_2445**	-6.1	DH	cytochrome P450. family 706 subfamily A. polypeptide 6	Oxidation-reduction
**c_7318**	-6.1	W	putative FKBP-type peptidyl-prolyl cis-trans isomerase 4	Protein folding
**c_3063**	-6.4	DH	Peptidyl-prolyl cis-trans isomerase CYP20-3	Protein folding
**c_12218**	-6.5	DH	CHAPERONIN 60 BETA (CPN60B)	Protein folding
**c_1608**	-6.8	DH	2-cys peroxiredoxin. chloroplast	Defense response
**c_4852**	-7	W	thioredoxin F-type 1	Cell redox homeostasis
**c_1814**	-7.2	W	phosphoethanolamine N-methyltransferase	Lipid biosynthesis
**c_280**	-7.9	D	epithiospecifier modifier	Glucosinolate biosynthesis
**c_469**	-8.5	D	oxalic acid oxidase	Nutrient reservoir activity

Abbreviations: C, cold; D, dark; DH, dehydration; W, wounding; S, salinity; H, heat; N-st, nitrogen starvation

**Fig 2:**
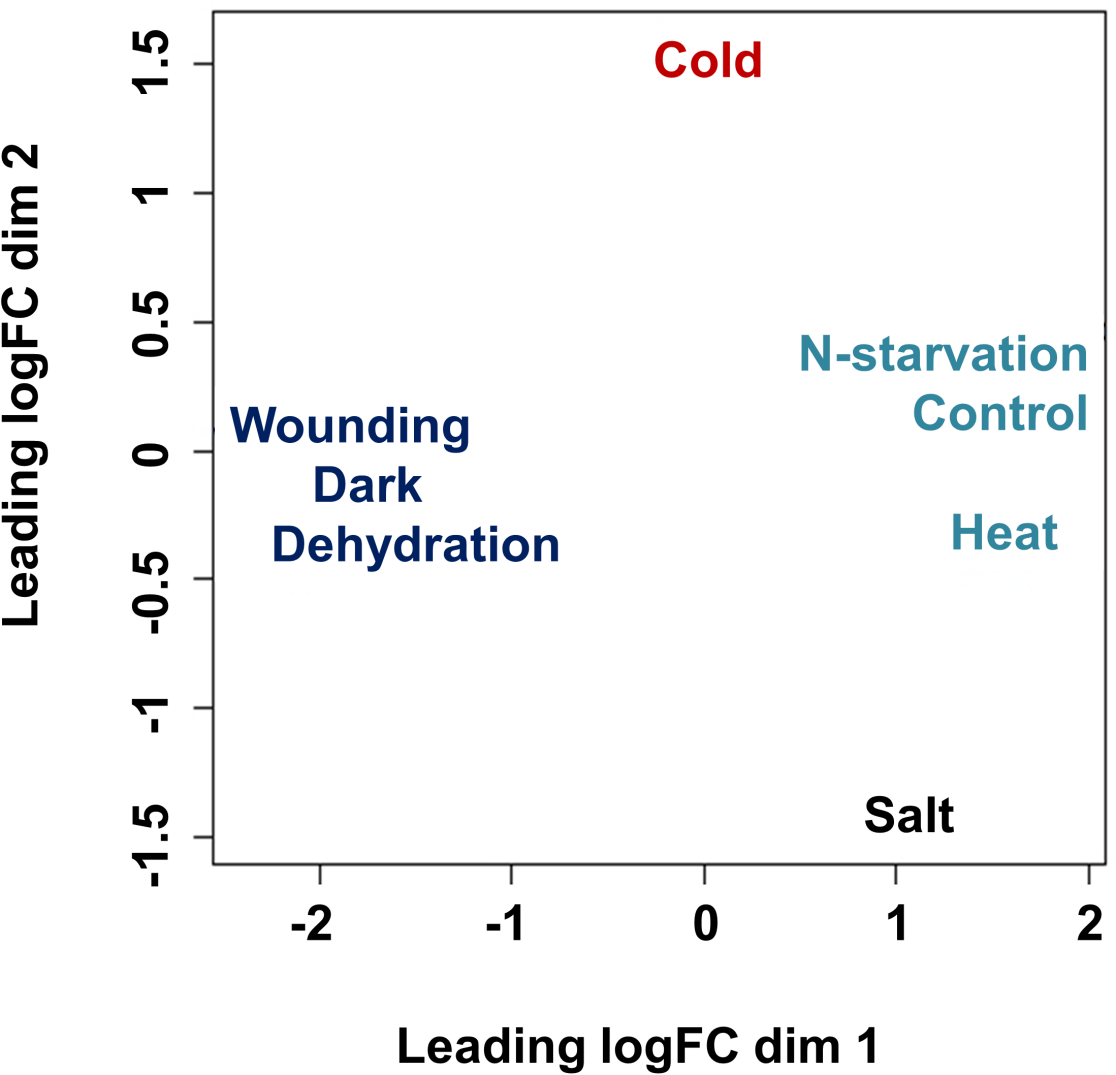
Multidimensional scaling (MDS) plots. The distances among DEGs correspond to leading log_2_FC between each pair of samples. Postharvest treatment libraries appear to be more heterogeneous than pre-harvest libraries that are clustered with control.

A significant number of genes matching to unknown proteins were differentially expressed, representing rocket specific postharvest stress-dependent genes. In most stresses more genes were down-regulated than up-regulated. Three postharvest treatments (wounding, dehydration and dark) clustered separately from the others on a multidimensional scaling (MDS) plot (Fig 2).

**Fig 3:**
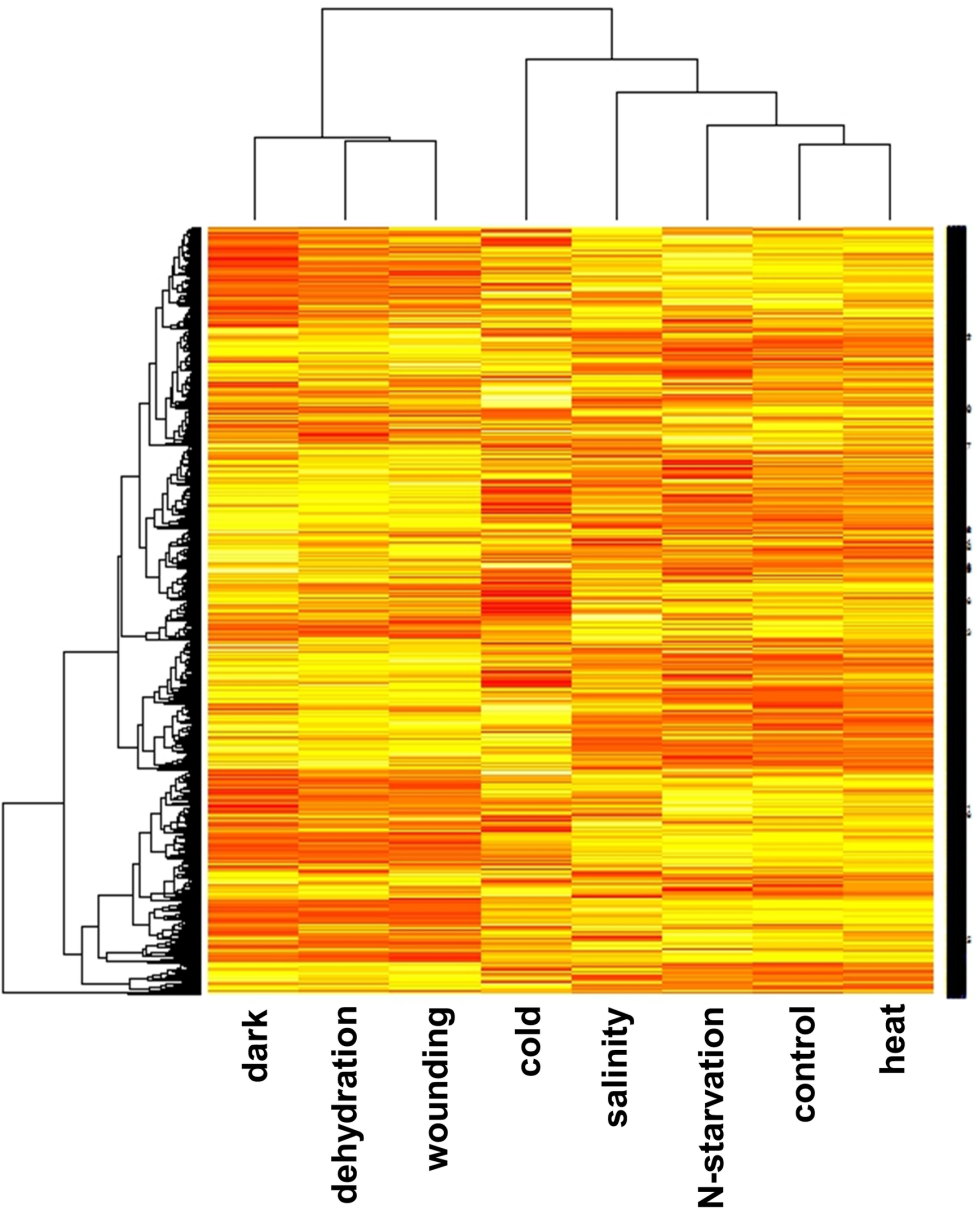
Heatmap obtained with edgeR. Dendrograms of the expression patterns were generated for each transcript in control and stress conditions. The bar colour reflects the gene expression levels as TMM normalized counts per million (CPM). Colour key indicates the intensity associated with normalized expression values. Dark and light shades indicate higher expression and lower expression respectively.

Cold and salinity each formed an independent group, while pre-harvest stresses (N-starvation and heat) grouped with the control indicating that the leaf transcriptome was not severely affected. Blocks of genes co-varied across dark, dehydration and wounding treatments (Fig 3).

Consistent with the MDS plot, gene expression under N-starvation and heat stress was similar to the control, whereas cold and salinity patterns differed from the control and all other stresses. MA-plots indicate that there was no systematic bias in the sequencing data (S4 Fig).

To validate the RNA-seq data, we selected 18 differentially expressed genes and assessed their expression level by real-time RT-PCR. RT-PCR reactions were performed on different RNA samples, that were collected from rocket plants grown and treated under the same stress conditions as those applied for the RNA-seq experiments.

In these samples, 13 out of 18 (72%) genes showed a similar trend to the RNA-Seq data (S5 Fig), thus the level of agreement between the RNA-Seq and real time PCR methods is indicative that the system was robust. A similar degree of agreement was seen in comparisons of microarray and RNA-Seq experiments in *Arabidopsis* indicating that this level of discrepancy is not unusual when using different methods for the analysis of gene expression [47].

GO categories corresponding to cellular and metabolic processes were the most enriched for almost all stresses (S4H Table; S6 Fig). Enriched KEGG pathways among DEGs were carbon metabolism and biosynthesis of amino acids, photosynthesis and hormone signal transduction (Table 7; S5 Table). Pathway enrichment analyses of DEGs were complemented with manually-based searches of genes related to leaf metabolism and stress responses (S6 Table). The number of DEGs was very limited under pre-harvest stress and no enrichment pathways were found.

**Table 7:** Enriched KEGG pathways of DEG in each stress condition.

*KEGG pathway*	C	D	DH	W	S	H	N-St.
01200 Carbon metabolism	11	33	34	33	10	-	1
01230 Biosynthesis of amino acids	13	42	56	53	14	3	-
00500 Starch and sucrose metabolism	11	14	20	20	7	-	-
00520 Amino sugar and nucleotide sugar metabolism	11	12	22	20	5	-	-
04075 Plant hormone signal transduction	21	14	23	22	9	4	-
00195 Photosynthesis	8	23	23	24	1	-	1
00564 Glycerophospholipid metabolism	10	2	12	12	-	-	-
00280 Valine, leucine and isoleucine degradation	10	15	15	16	4	1	-
00330 Arginine and proline metabolism	10	14	17	17	5	1	-
01210 2-Oxocarboxylic acid metabolism	3	17	19	19	6	1	-
01212 Fatty acid metabolism	5	13	15	16	5	-	-
00010 Glycolysis / Gluconeogenesis	6	13	16	15	6	1	-
00030 Pentose phosphate pathway	1	7	10	8	2	-	-
00051 Fructose and mannose metabolism	2	7	11	8	1	-	-
00053 Ascorbate and aldarate metabolism	4	6	10	9	3	1	-
00620 Pyruvate metabolism	6	10	9	10	7	1	-
00630 Glyoxylate and dicarboxylate metabolism	6	14	14	15	6	1	1
00190 Oxidative phosphorylation	7	12	9	8	-	-	-
00196 Photosynthesis—antenna proteins	3	9	11	11	-	-	-
00710 Carbon fixation in photosynthetic organisms	3	17	16	17	3	-	1
00071 Fatty acid degradation	7	8	9	10	3	1	-
00561 Glycerolipid metabolism	9	7	13	13	4	2	-
00230 Purine metabolism	6	12	30	28	4	1	-
00240 Pyrimidine metabolism	7	5	21	19	6	1	-
00250 Alanine, aspartate and glutamate metabolism	6	10	17	15	2	-	-
00260 Glycine, serine and threonine metabolism	6	11	15	16	4	1	-
00270 Cysteine and methionine metabolism	3	11	11	13	4	1	-
00350 Tyrosine metabolism	4	6	8	10	4	1	-
00360 Phenylalanine metabolism	6	8	7	10	5	2	1
00860 Porphyrin and chlorophyll metabolism	4	16	19	21	2	1	-
00900 Terpenoid backbone biosynthesis	6	7	10	7	2	1	-
03008 Ribosome biogenesis in eukaryotes	2	1	12	13	1	-	-
04141 Protein processing in endoplasmic reticulum	12	18	14	14	4	-	-
04120 Ubiquitin mediated proteolysis	6	8	11	14	5	-	-
03030 DNA replication	2	2	15	10	2	-	-
04144 Endocytosis	6	2	9	12	3	-	-
04142 Lysosome	3	9	11	12	2	-	-
04146 Peroxisome	6	9	14	15	5	1	-
04110 Cell cycle	3	3	16	13	4	-	-

Abbreviations: C, cold; D, dark; DH, dehydration; W, wounding; S, salinity; H, heat; N-st, nitrogen starvation

### Genes related to plant hormones

**Fig 4:**
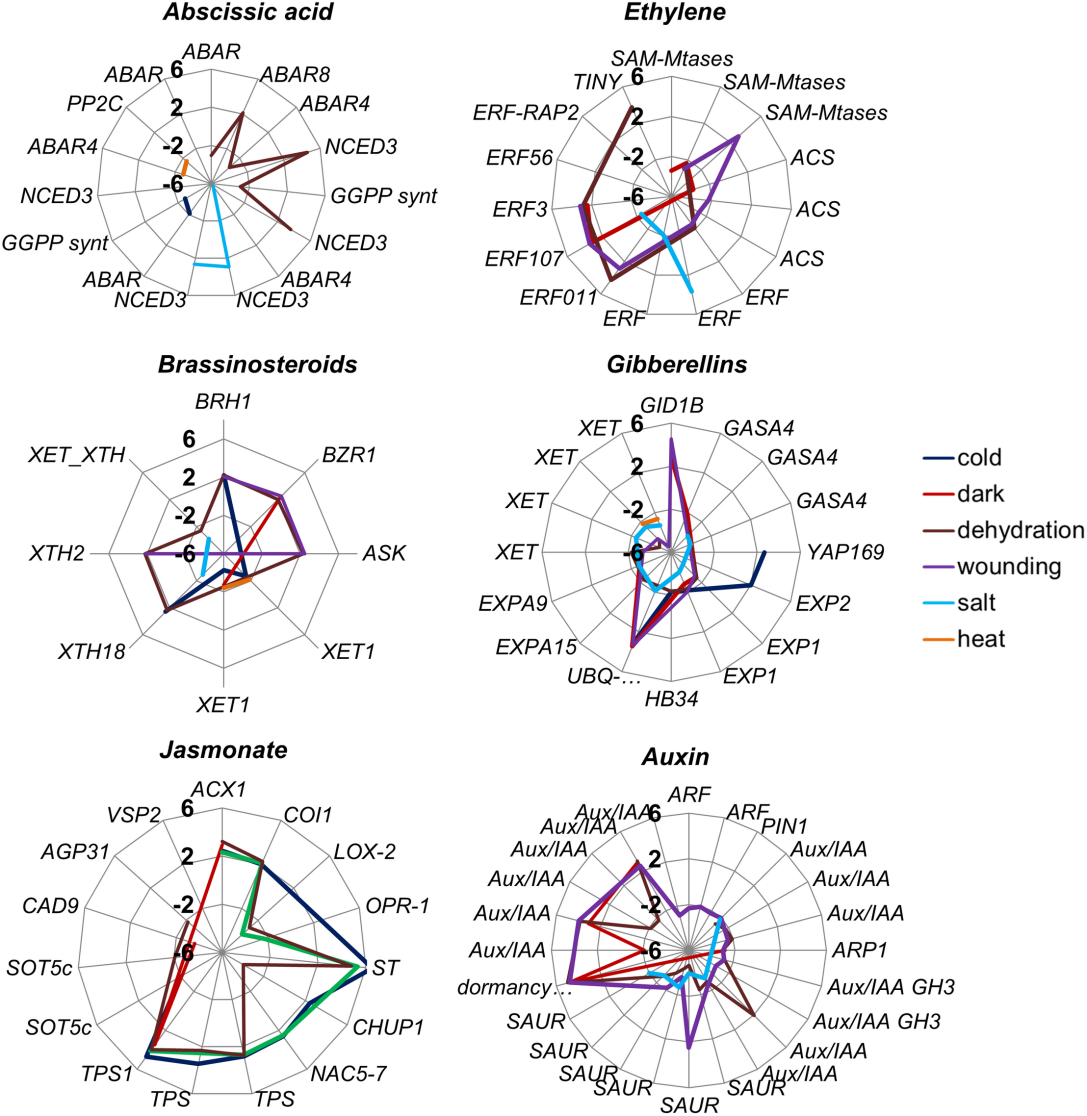
Radar graph representing differentially expressed genes involved in hormone biosynthesis and response. Each concentric ring represents log_2_FC increase or decrease, where axes represent maximum FC = +6 (outer ring), minimum FC = –6 (central point) and zero as no change. Stress treatments are presented by different colors.

Thirty-one contigs involved in ABA biosynthesis, perception and catabolism were identified (Fig 4, S6A Table).

Only a small number of ABA-related genes changed significantly in expression following each stress treatment: the highest number (six) in response to dehydration as might be expected, two in response to cold and one in response to wounding. Two genes with homology to *NCED3*, a key gene in ABA biosynthesis, were highly up-regulated following dehydration, salinity and dark treatments. Genes encoding ABA receptors or components of the ABA receptor complex (*ABAR*, *ABAR4* and *PP2C)* were generally down-regulated by the treatments although the regulation may be complex since three DEGs with homology to *ABAR* and *ABAR4* showed different expression patterns. However, *PP2C* was only up-regulated by heat.

As expected the highest number of genes related to JA (ten) responded to wounding followed by cold (nine), dehydration (eight) and dark (three). Twenty eight genes encoding enzymes involved in JA-biosynthesis were identified including lipoxygenase (*LOX*), allene oxidase cyclase (*AOC*), 12-oxophytodienoate reductase (*OPR*), peroxisomal acyl-CoA-oxidase 1 (*ACX1*), 12-oxophytodienoate reductase 1 (*OPR1*), jasmonic acid-amido synthetase (*JAR1*), coronitine insensitive 1 (*COI1*) and 2 carboxylmethyltransferase (*JMT*). *LOX2* and *OPR1* involved in JA biosynthesis were down-regulated while *ACX1* also involved in JA biosynthesis and *COI1* involved in JA responses were up-regulated in response to dehydration and wounding.

Wounding elicited most change in the expression of genes related to ethylene (eight) followed by dehydration and dark (five respectively). Four S-adenosyl-L-methionine synthetase (*SAM*), three 1-aminocyclopropane-1-carboxylic acid (*ACC*) synthase genes (*ACS*) and nine ACC oxidase (*ACO*) functioning in ethylene biosynthesis were identified, but only *SAM* and *ACS* genes were down-regulated under dark and wounding. Among the 26 ethylene responsive factors, *ERF3*, *ERF011/CEJ1* and *ERF107* were up-regulated following postharvest stresses and only *ERF039* following salinity. Five ethylene insensitive genes were identified but only *EIN3* showed up-regulation following postharvest stresses along with ethylene-responsive TFs *RAP2-3* and *TINY*.

Of the more than 100 transcripts implicated in auxin biosynthesis, transport and metabolism, 38 were affected by postharvest stresses, mostly by dehydration or wounding (17 and 16 respectively), while five were affected by dark. Twelve auxin biosynthetic genes (including probable indole-3-pyruvate monooxygenase (*YUCCA9*), nitrilase (*NIT*), amidohydrolase genes (*IAR*) and indole-3-acetic acid-amido synthetase (*GH3*.*5*)) were identified. Of the 60 transporters (including auxin influx carrier (LAX), efflux carrier component (PIN) and p-glycoproteins and SAUR-like transporters (SAUR)) only six were down-regulated under dehydration and salinity. Among auxin responsive genes (21 ARF, 19 Aux/IAA and three dormancy/auxin associated genes), eight were differentially expressed. Most auxin-related genes were down-regulated under dark, dehydration and wounding. However, the expression of dormancy/auxin associated gene *DRM* was up-regulated under wounding, dark and dehydration stresses.

Surprisingly, expression of a large number of GA-related genes (25) was altered by the stresses. In contrast, the expression of GA biosynthetic genes was unaffected. Only gibberellin receptor 1B (*GID1B*) was up-regulated in response to dark, dehydration and wounding.

Expression of 16 brassinosteroid-related genes was altered in postharvest: most by dehydration (six), cold (four) and wounding (four), while only two by dark. Three transmitters of the BR signal (shaggy-related protein kinase (*BIN2*), brassinazole resistant (BZR) and brassinosteroid-responsive RING-H2 (BRH1) were up-regulated following postharvest stresses as well as target genes including members of the xyloglucan endotransglycosylase (XTH) and xyloglucosyl transferase (XET) families.

### Regulation of gene expression

**Fig 5:**
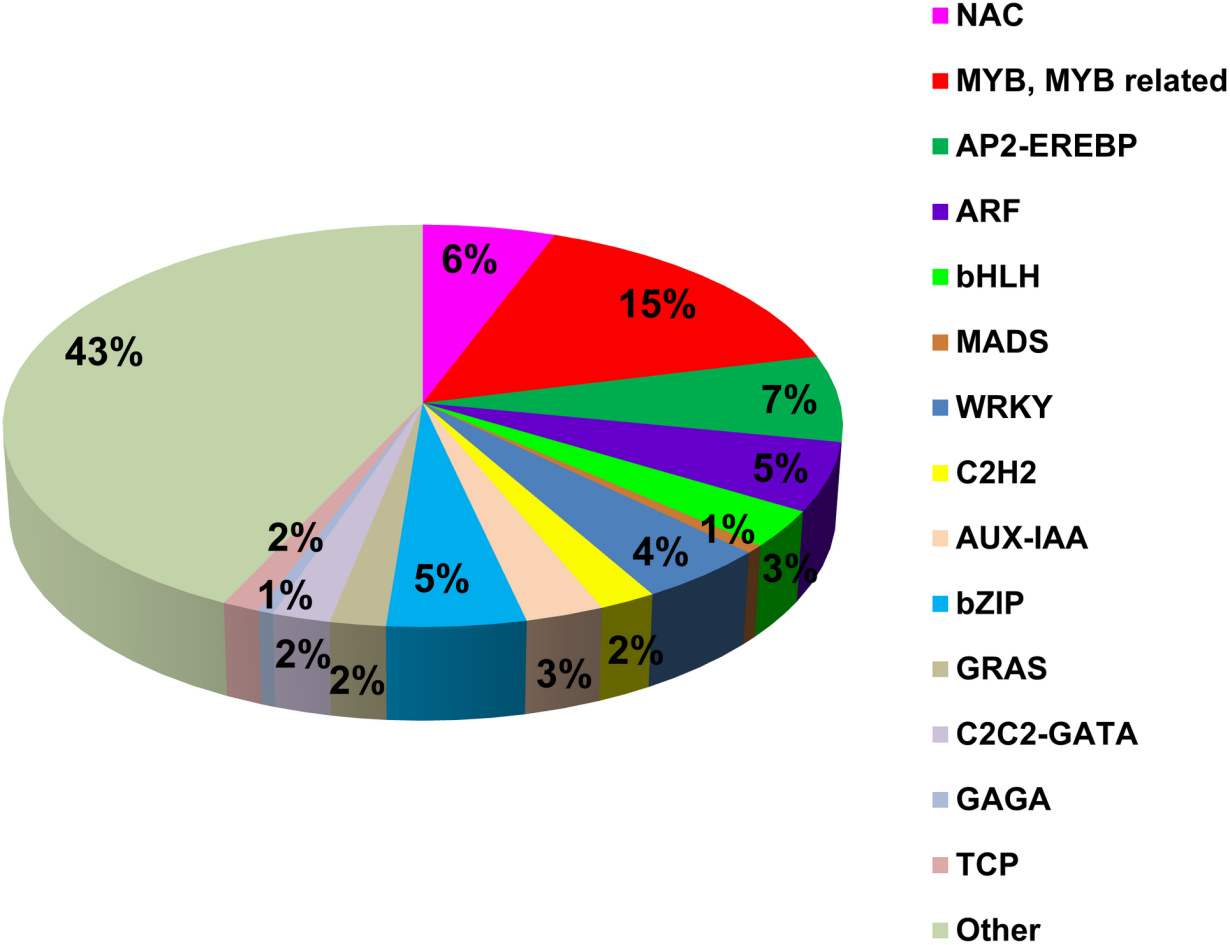
Most represented transcription factor families in the transcriptome. The % of each family as a proportion of all the TFs annotated in the transcriptome.

Of the 2125 genes with homology to TFs, 57% were clustered into families, mostly represented by the *MYB*/*MYB* related (15%), *AP2-EREBP* (7%), *NAC* TFs (6%) and both *ARF* and *WRKY* (5%) (Fig 5).

**Fig 6:**
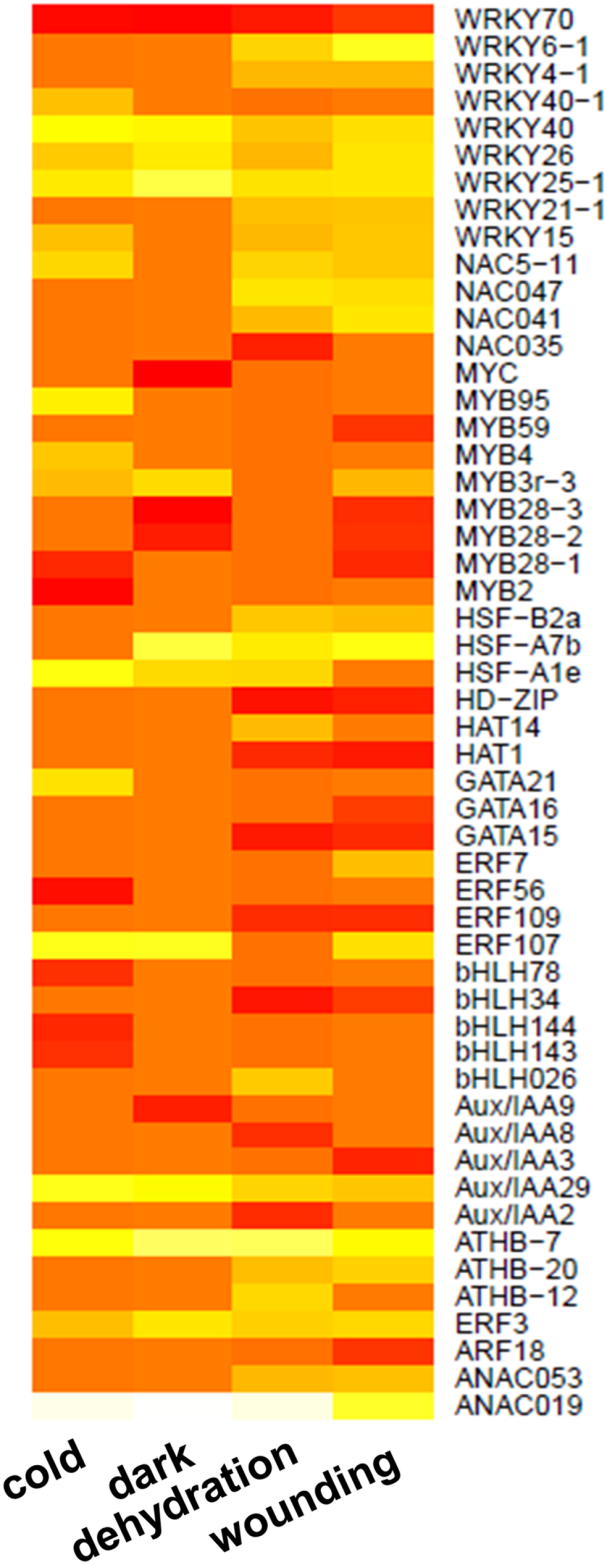
Differentially expressed genes encoding transcription factors. Heatmap of the log_2_FC calculated with edgeR. The bar colour reflects the FC levels. Dark and light shades indicate lower FC and higher FC respectively.

Among them, 735 DEGs were found across all stresses (Table 8). More genes were up-regulated than down-regulated in most stresses and largely belonged to the *WRKY* and *MYB* families across all treatments. Other TF families highly associated with stress responses belonged to *AP2-EREBP*, *AUX-IAA*, *NAC*, *bZIP* and *bHLH* families. Some co-expression of TFs was seen across all postharvest treatments. For example *WRKY70* expression was highly down-regulated and *ANAC059* was up-regulated across all treatments. Expression of other TFs was more specific to combinations of stresses (Fig 6A; S6B Table).

**Table 8:** Differentially expressed transcription factors.

Treatment	Up-regulated genes	Down-regulated genes	Most represented TF families
Cold	115	30	bHLH, WRKY, MYB, AP2, bZIP
Dark	63	23	MYB, NAC, WRKY, AP2
Dehydration	123	88	NAC, MYB, WRKY, AUX-IAA
Wounding	120	90	AP2, WRKY, MYB AUX-IAA, bZIP
Salinity	29	44	NAC, AP2, bZIP
Heat	1	9	AP2, zf-C2H2, GATA
N-starvation	-	-	-
Total	451	284	

Total number of DEGs encoding for transcription factors at FDR ≤ 0.05 according to FC ≥ 2 and FC ≤ -2 for up-regulated and down-regulated genes respectively.

Wounding stimulated expression of *MYB51* and *MYB51-2*, which control the biosynthesis of indole glucosinolates, while *MYB28* was repressed. Amongst the *WRKY* TFs *WRKY6*, *WRKY4* and *WRKY11* were up-regulated by wounding, while cold only up-regulated *WRKY11*.

*ERF3* was among the most expressed genes following dehydration, dark and cold stress, while *RAP2*.*3* and *RAP2*.*9* homologous to the *RAP2* sub-family of *ERF/AP2* TFs, were among those whose expression was most affected by cold. *RAP2*.*3* was also highly expressed in response to dehydration. Among the *NAC* TFs, *NAC019* and *NAC059* were up-regulated in response to five of the stresses: dehydration, wounding, dark, cold and salinity, while expression of *NAC053* and *NAC052* was up-regulated only in response to dehydration, wounding and dark, and rocket *NAC51* was only up-regulated by dehydration and wounding.

### Genes related to ROS homeostasis, autophagy and senescence

There was no up-regulation in the expression of genes for ROS detoxification including 23 cytochrome c oxidases and 18 NADH-ubiquinone oxidoreductases. On the contrary, *SOD*, *APX* and *GPX* were down-regulated. Only *CAT* expression increased suggesting induction of a compensatory mechanism to balance the reduced expression of *SOD*, *APX* and *GPX*. Other genes in the ascorbate–glutathione pathway for ROS scavenging (monodehydroascorbate reductase (*MDHAR*), dehydroascorbate reductase (*DHAR*) and glutathione reductase (*GR*)) did not vary in their expression levels. However, some genes involved in the biosynthesis of non-enzymatic antioxidants such as tocopherol and alkaloids (e.g 4-hydroxyphenylpyruvate dioxygenase (*HPPD*), strictosidine synthase (*STR*) were strongly up-regulated following postharvest treatments, although other tocopherol biosynthetic genes such as tocopherol cyclase (*VTE1*) were down-regulated.

Twenty four autophagy-related genes (*ATG*) were identified including components of the ubiquitin-like conjugation systems (*ATG3;* three *ATG4;* ten *ATG8* genes), the TOR kinase signaling pathway (four *TOR* genes), autophagosome formation (*ATG5*), the *ATG1* kinase complex (*ATG13*) and membrane cycling (*ATG2; ATG18; ATG9*). Two *ATG8a* genes, *ATG8e*, *ATG13*, *ATG4* and *ATG2* were up-regulated by the stress treatments indicating activation of autophagy processes. Three *HVA22A* genes with putative roles in autophagy were highly expressed under postharvest stresses and salinity. Interestingly, ten sequences matching hypothetical or uncharacterized proteins mapped to autophagy-related GO terms and were up-regulated by dehydration and wounding (S6C Table).

In response to the stresses, photosynthetic genes such as ribulose bisphosphate carboxylase (*Rubisco*) and thirteen chlorophyll a/b binding proteins (CAB) were strongly down-regulated. A magnesium protoporphyrin IX gene and a member of the *FtsH* metalloprotease family, both involved in photoprotection but also in chloroplast biogenesis, were down-regulated implying a decrease in the biosynthesis of chlorophyll and chloroplast biogenesis. In contrast, genes encoding enzymes responsible for the degradation of plastid proteins (plastidic metallo-beta-lactamase and eight plastid CLP-protease genes) and chlorophyll degradation (pheophytinase (*PPH*) and pheophorbide a oxygenase (*PaO*)) were up-regulated. Interestingly, the Circadian clock-associated 1 (*CCA1*) gene was up-regulated only under cold, while phytochrome A (*PHYA*), phytochrome E (*PHYE*) and phytochrome C (*PHYC*) did not vary.

Several genes involved in protein (e.g cysteine proteases, ubiquitin, RING finger proteins) and lipid (lipase, esterase, 3-hydroxyacyl-CoA dehydrogenase) degradation were up-regulated but not genes involved in polysaccharide breakdown. However, note that no homologue of *SAG12* was found in the rocket transcriptome.

### Glucosinolate (GSL)-related genes

A total of 179 sequences involved in GSL metabolism were identified including biosynthesis (side-chain elongation of amino acids, development of the core structure, and secondary side-chain modifications) and catabolism genes. Wounding elicited most DEGs (36) followed by dehydration (25), dark (21) and cold (12) (S7 Fig; S6D Table). Pre-harvest treatments poorly affected the expression of GSLs-related genes. Postharvest stresses seemed to induce the metabolism of indole GSLs rather than GSLs derived from other amino acids: for example cold up-regulated an *O*-methyltransferase family 2 gene involved in the biosynthesis of indole glucosinolates. Most of the GSL-related sequences up-regulated under dehydration did not return a blast hit, but GO annotation assigned them to indole glucosinolate biosynthetic process. Many down-regulated genes were involved in the aliphatic and in the main glucosinolate biosynthetic pathway (e.g. *MAM1*, *CYP79F1*, *CYP83A1*, *ST5c*). Sequences with high similarity to sulfotransferase 5c (*ST5c*) and methylthioalkylmalate synthase 2 (*MAM2*) were also repressed by cold. Similarly, under dark, *O*-methyltransferase family 2 and genes involved in GSL biosynthesis such as 3-isopropylmalate dehydratase, *CYP79F1* and *CYP83A1* were down-regulated. However, thiocyanate methyltransferase 1 (*TMT)1* involved in GSL catabolism was up-regulated in wounded, cold and dark-stressed leaves.

## Discussion

The goal of this study was to reveal the molecular mechanisms of stress responses in rocket through the identification of genes and pathways activated pre- and postharvest. Although pre-harvest and postharvest stresses contribute to postharvest quality losses [48], gene expression changes elicited by pre- and postharvest treatments in rocket leaves were clearly separated. This may be due to the way the treatments were applied which was consistent with the ways these stresses would be imposed in the industry. Postharvest stresses were applied to the harvested leaves, while pre-harvest stresses were applied via the nutrient solution, hence roots were affected most directly. This could explain the lower number of DEGs found following N-starvation and heat.

**Fig 7:**
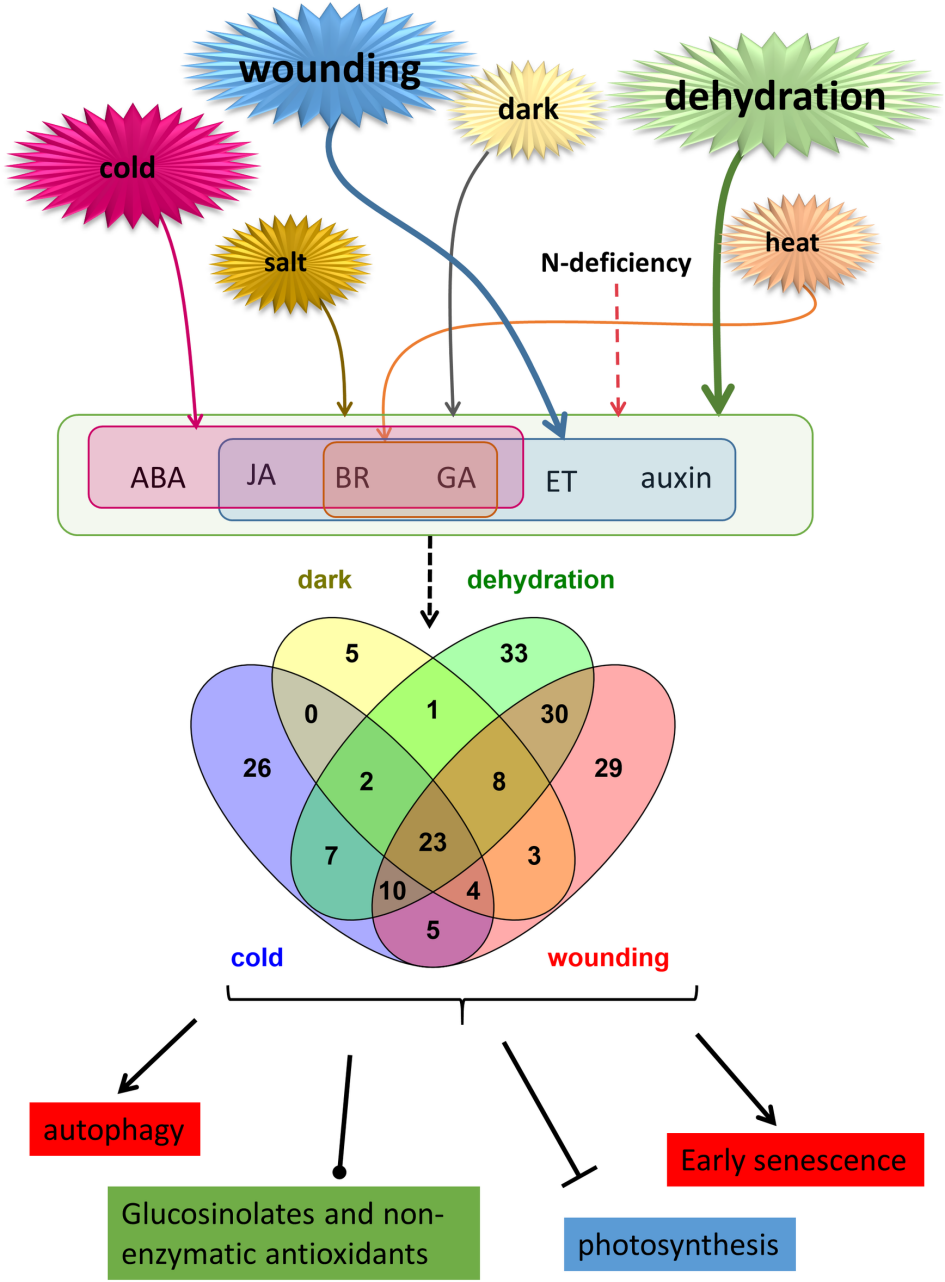
Model illustrating the overlapping effects of 24h stress treatments on PGR signaling, TF expression and down-stream processes. Stress font size indicates the importance of each stress in altering gene expression. Shaded Venn diagram circles indicate number of TFs from the major families identified from the transcriptome (MYB, WRKY, bHLH, AP2, bZIP, NAC, AUX-IAA, Zf-C2H2, GATA and homeobox domain) whose expression changes in response to overlapping postharvest stresses.

The pattern of DEG expression provides some insights into the regulation of postharvest stress responses in rocket (Fig 7).

The activation of overlapping sets of PGR-related genes fits with our understanding of the networked regulation of leaf stress responses [49]. The identification of overlapping sets of TFs activated will provide further scope for understanding the postharvest TF network that is emerging for senescence and abiotic stresses [50].

Although comparisons can be made to detached leaf studies in *Arabidopsis* and other species [5,51], this is the first study to compare such a wide range of postharvest stresses. Furthermore, all postharvest treatments were conducted in the dark and thus are in fact imposing a combined stress. This adds extra complexity as different and opposing responses may be elicited making the outcome, here assessed as gene expression changes, difficult to predict [52,53]. For example, dehydration and wounding generated the greatest responses, whereas in previous studies comparing multiple stresses over a 24h time-period [2], cold and salinity treatments elicited the most changes compared to drought, heat and wounding, although the latter stresses were milder than those used here. This suggests that in rocket minimal processing and retaining humidity during processing are key factors in minimizing stress to the leaves.

Dehydration and wounding altered the highest number of PGR-related genes and genes related to the highest number of different PGRs, indicating multiple mediators of the stress in downstream events. However, few ABA-related transcripts were affected by wounding and few auxin and ethylene-related transcripts were affected by cold. This is consistent with the complex crosstalk between PGRs in stress responses [14,6] and indicates that manipulating PGRs for improving stress resilience would need to be tailored to particular stresses or combinations of stresses.

Fine-tuned control is also an important factor. For example JA biosynthesis genes were mostly down-regulated, except *COI1* which was up-regulated in response to wounding, consistent with its role in *Arabidopsis* in mediating wound-induced JA dependent responses [9]. However, *OPR1* was down-regulated in rocket leaves 24h after wounding, while in Arabidopsis *OPR1* was only transiently up-regulated with a peak at 3-6h [54].

This complex temporal control may also explain some of the changes seen in genes related to auxin. Most auxin-related genes were down-regulated in rocket under dark, dehydration and wounding. However, in *Arabidopsis* wounding down-regulated auxin-responsive genes over a 6h time-frame [11]. This difference in timing may be due to the combined wounding/dark stress treatment. Since wounding elicits ethylene production, this may also be another example of the coordination between the two PGRs based on a common precursor for their biosynthesis [55]. Crosstalk of auxin with ethylene and ABA is found through TFs that respond to combinations of these PGRs [6]. The down-regulation of putative gibberellin target genes such as gibberellin-regulated protein 4 (*GASA4*), xyloglucan endotransglycosylase (*MERI5*) and expansin (*EXP*) involved in cell wall weakening under several stresses is consistent with an inhibition of growth during stress mediated by GA and auxin. The up-regulation of BR signaling following postharvest stresses adds further support to the role of BRs in protecting against abiotic stress [13], probably mediated through ROS [56].

Although expression of some rocket ROS-related enzymes was unaffected by the stresses, *CAT* expression was up-regulated indicating possible changes in ROS-response. Unchanged expression levels of the ascorbate–glutathione pathway and up-regulation of some tocopherol and alkaloid biosynthetic genes indicate that in rocket the latter molecules may be the prevalent antioxidant mechanisms under short-term stress. Hence, increasing their levels may improve stress resilience in rocket at this stage.

Changes in the expression of combinations of TFs were elicited by the different stresses with the transcription of the *MYB* and *WRKY* TFs activated by all the postharvest stresses. This suggests that manipulation of *MYB* and/or *WRKY* TFs might be a promising avenue for modulating postharvest stresses in rocket. The subfamily of *R2R3-MYB* proteins are key regulators of *Arabidopsis* stress responses [57]. However, in rocket their expression pattern was complex and stress-specific. *MYB4* was induced by cold, but expression of *MYB2* and *MYB28-1* was down-regulated. *R2R3-type MYB* genes regulate several aspects of plant secondary metabolism [58]. In *Arabidopsis MYB4* is involved in stress responses mediated by flavonoids [59], while *AtMYB2* regulates *AtADH1* (alcohol dehydrogenase1). Furthermore, *MYB28* is a key regulator of aliphatic glucosinolate metabolism [60]. Since changes in rocket *MYB* gene expression paralleled changes in expression of glucosinolate-related genes, these *MYB* TFs may play a role in the changes in concentrations of glucosinolates and other secondary metabolites in rocket as seen in other brassicas under stress [61]. These *MYB* TFs may therefore be useful targets for breeding rocket with improved glucosinolates and flavonols which is of commercial interest [62].

Based on *Arabidopsis*, regulatory relationships can be inferred for the expression of the ten *WRKY* genes in rocket. The expression of *WRKY70* and *WRKY6* following dehydration and wounding is consistent with the proposed regulatory loop in *Arabidopsis* in which *WRKY70* down-regulates *WRKY6* via the up-regulation of *WRKY53* [63], although *WRKY53* was not detected among the rocket DEGs. In *Arabidopsis WRKY40* is implicated in ABA responses: ABA binds to this TF removing it from the nucleus and releasing its inhibition from ABA-responsive genes [16]. Hence, the up-regulation of *WKY40* in all rocket stress treatments may indicate a role in mediating ABA responses. Three of the *WRKY* TFs in the rocket DEGs (*WRKY11*, *WRKY25* and *WRKY40)* are included in the N12 cluster of universal stress response genes [64] in *Arabidopsis* roots. N12 cluster genes are all ABA regulated and may contribute to ABA responses in rocket. However, rocket *WRKY11* was only up-regulated by wounding and down-regulated by cold, indicating that its regulation may be more stress-specific. In *Arabidopsis*, expression of 17 *WRKY* TFs appear to be ROS regulated [17]. Of these *WRKY6*, *WRKY15*, *WRKY22* and *WRKY25*, *WRKY40* and *WRKY 70* are represented in the rocket DEGs suggesting a role in mediating ROS-induced stress responses. Overall, these data suggest that the regulatory *WRKY* TF network proposed for *Arabidopsis* [65] may also be broadly relevant to postharvest stresses in rocket, but needs to be investigated in more detail.

*ERFs* were induced by all postharvest stresses as well as heat and salinity indicating a broad role in rocket stress responses. Again, some gene responses were shared with *Arabidopsis*, however the timing of the response differed. The rocket *ERF3* was highly up-regulated by several stresses after 24h. In contrast, in *Arabidopsis* it is very transiently up-regulated in response to whole plant abiotic stresses and by 24h the expression returned to the initial levels [2]. This suggests that in rocket leaves the postharvest response to ethylene may be slower and more prolonged than the pre-harvest stresses in *Arabidopsis*. However, the expression patterns of *RAP2*.*3* and *RAP2*.*9* were more similar to those of the *Arabidopsis* homologues [66,67] indicating conserved function of these two genes.

*ANACO19*, which was strongly up-regulated in response to all the rocket postharvest stresses, has been identified as an important hub in abiotic stress responses [17] and senescence in *Arabidopsis* [68]. *AtANA019* acts via a range of other TFs and via ABA, JA and SA, and may regulate flavonoids in response to drought and salinity. *AtANA019* is in turn regulated by *MYB2* [68], another gene found among rocket DEGs. However, in rocket *MYB2* was only down-regulated in response to cold, therefore its interaction with *ANAC019* may be stress-specific. A full understanding of gene regulation involving rocket *ANACO19* in postharvest may provide useful targets for breeding and biotechnological approaches to improving postharvest resilience.

Understanding the fine regulation of rocket *ANAC053* may also be important in postharvest management as *AtANAC053* regulates heat-stress induced programmed cell death (PCD) via a ROS signal [69]. Although autophagy-related genes and plastid disassembly related genes were amongst the rocket DEGs, *SAG12*, which is an accepted marker of leaf senescence, was not up-regulated. This suggests that senescence and PCD are likely to be at a very early stage after the 24h stress treatments, although they may be accelerated later in the postharvest storage period. In contrast, the stresses appeared to stimulate catabolism of GSLs. This would lead to the formation of important health-related compounds (isothiocyanates and thiocyanates), but this benefit needs to be carefully balanced against a loss of quality through premature deterioration of the leaves.

This study has significant implications in the postharvest field. Many seed companies are including rocket species in breeding programmes for developing cultivars with improved shelf-life [70]. Our RNA-Seq analysis provides a rich and new transcriptomic resource that has been already exploited to develop quality markers for rocket [71] and can be used therefore in conventional and biotechnological breeding approaches. The latter will need to be combined with physical and chemical postharvest treatments in order to preserve quality and safety of fresh-cut produce.

## Supporting information

S1 Text *De novo* assembled transcript sequences in FASTA format. (7Z)

S1 Table List of primers used in real time RT-PCR for the validation of differentially expressed genes across pre- and postharvest stress treatments. (XLSX)

S2 Table *De novo* transcriptome assembly statistics. S2A Table reports the length (bp) for each contig, the number of reads that entered into the contig assembly (total read counts), the number of reads that entered into the contig assembly as single read (single reads) and as paired-end (reads in pairs) and the average coverage. S2B Table reports the total number of reads (unique gene reads) that were mapped uniquely to one place in the reference sequence, the number of total reads and the RPKM values for each contig.
(XLSX)

S3 Table Functional annotation of the rocket leaf transcriptome. S3A Table reports the results from FastAnnotator analysis: blast hit score, gene ontology mapping, enzyme commission numbers and pfam domains for the 33874 transcripts. S3B Table reports the results of the slim classification of the Gene Ontology terms by using CateGOrizer. S3C Table reports the results from the KAAS analysis against the KEGG GENES database and shows the number of contigs that is found in each KEGG pathway. S3D Table reports the list of KO assignments based on the bi-directional best hit of BLAST. S3E Table reports the BRITE hierarchical list of the annotated genes according to the BRITE database.
(XLSX)

S4 Table Differential expression analysis of rocket transcripts under stress treatments compared to the control. S4A, S4B, S4C, S4D, S4E, S4F and S4G Table reports the list of differentially expressed genes under cold, dark, dehydration, wounding, salinity, heat and N-starvation stress respectively using as threshold FDR ≤ 0.05 and FC ≥ ±2 (highlighted in yellow). S4H Table reports the "GO_Slim" classification results of DEGs in each stress by using the CateGOrizer tool.
(XLSX)

S5 Table Pathway enrichment analysis of differentially expressed genes in each stress compared to the control. S5A, S5B, S5C, S5D, S5E, S5F, S5G Table reports the list of enriched pathways obtained with KAAS according to the KEGG BRITE database (KEGG modules) under cold, dark, dehydration, wounding, salinity, heat and N-starvation stress, respectively. S5H and S5I Table report a summary of number of DEGs found in each pathway in postharvest and pre-harvest stresses, respectively.
(XLSX)

S6 Table Output results of manually-based searches of differentially regulated genes. Subset of DEGs (FDR ≤ 0.05; FC ≥ ±2) related to hormone biosynthesis (S6A Table), to transcription factors genes (S6B Table), to autophagy and senescence associated processes (S6C Table) and to glucosinolate metabolism (S6D Table). NS: Not Statistically Significant.
(XLSX)

S1 Fig BLAST results. A) Alignment distribution of blast hits. B) Numbers of top hit sequences from BLASTX calculated for each species. C) Species-based distribution of blast hits.
(TIF)

S2 Fig GO-level distribution. The graph shows the distribution of the Gene Ontology hits by GO level in Biological Process (P), Molecular Function (F) and Cellular Component (C). In total 5682 GO terms were distributed between level 2 and 12: 20002 sequences were involved in biological processes, with a peak at level 6, 21054 and 19508 transcripts had a biological function and cellular component respectively with a peak at level 7.
(TIF)

S3 Fig Most represented GO terms in each of the three GO root domains. From left to the right, the 5 GO terms with the highest counts in biological process, cellular component and molecular function are shown.
(TIF)

S4 Fig MA plots reporting the log-fold-(FC) changes against log-counts-per-million (CPM). Each plot shows the gene expression as logFC ratio versus abundance in CPM of each transcript for each treatment versus the control. Each dot represents a gene. Points representing significantly (FDR ≤ 0.05) differentially down-regulated and up-regulated genes are shown in red below -2 FC or above 2 FC respectively. Blue lines indicate a logFC = 1.
(TIF)

S5 Fig Real-time RT-PCR results. 18 genes were selected across the seven stressed libraries and real time RT-PCR performed as described in the methods section (mean ± S.E.; n = 3).
(TIF)

S6 Fig Gene ontology classes. The chart shows the most enriched GO slim terms for each stress.
(TIF)

S7 Fig Differentially expressed genes involved in glucosinolate metabolism for postharvest stresses. Heatmap of the log FC calculated with edgeR. The bar colour reflects the FC levels. Dark and light shades indicate lower FC and higher FC respectively.
(TIF)
