# Supplementary figures and images for: Gene expression analysis of rocket salad under pre-harvest and postharvest stresses: A transcriptomic resource for Diplotaxis tenuifolia
Source: PLoS One. 2017 May 30;12(5):e0178119. doi: 10.1371/journal.pone.0178119 (PMC5448768; doi:10.1371/journal.pone.0178119)

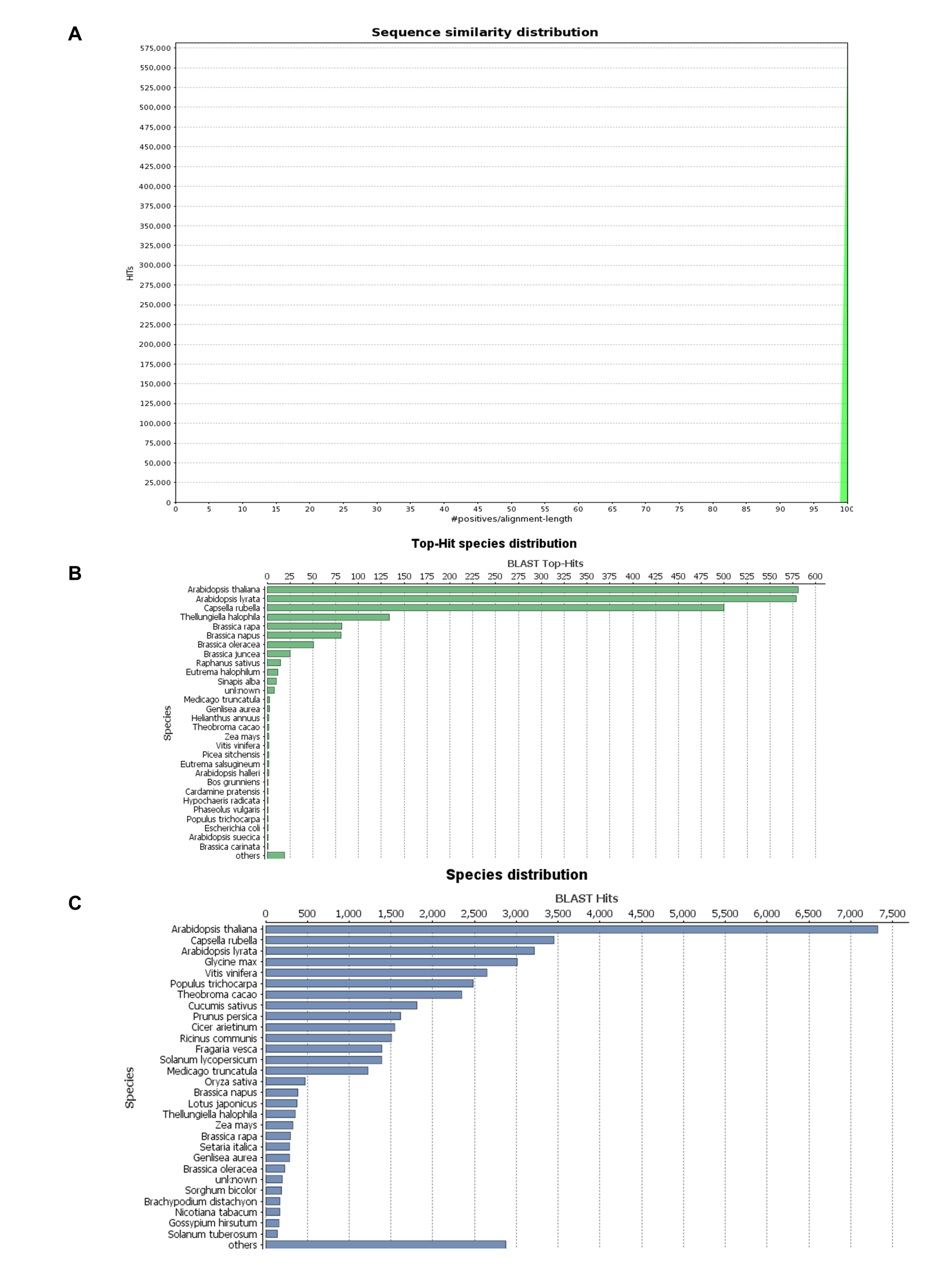

Supplement: S1 Fig — A) Alignment distribution of blast hits. B) Numbers of top hit sequences from BLASTX calculated for each species. C) Species-based distribution of blast hits. (TIF) [file pone.0178119.s008.tif]

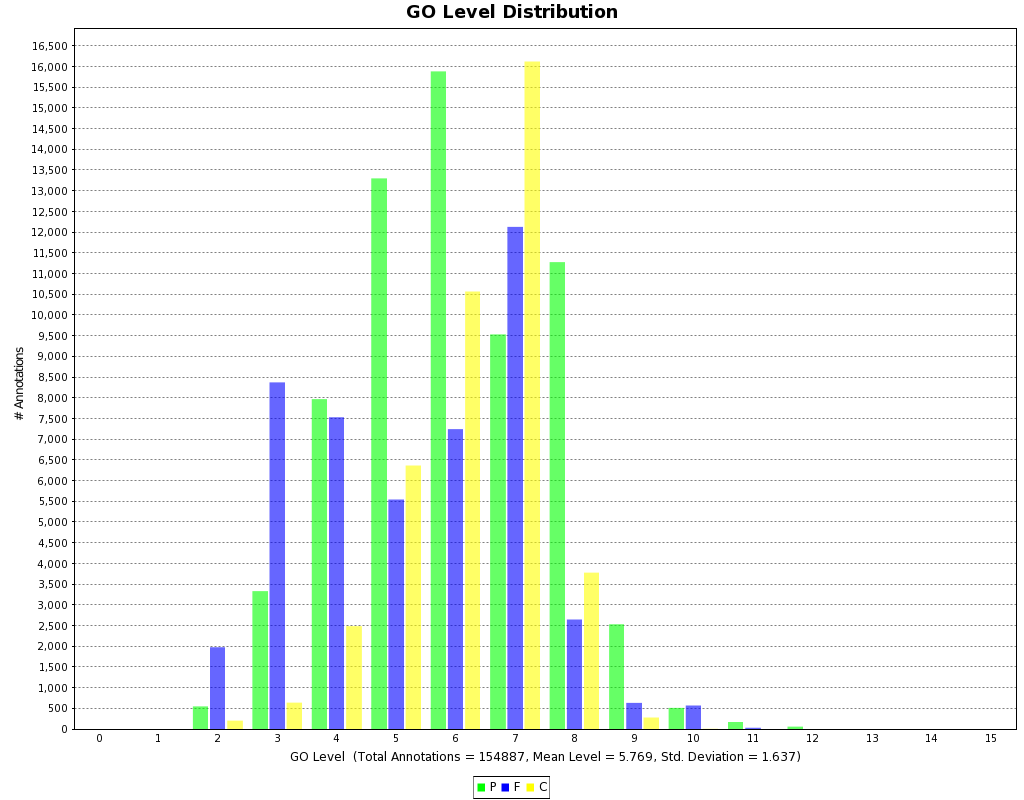

Supplement: S2 Fig — The graph shows the distribution of the Gene Ontology hits by GO level in Biological Process (P), Molecular Function (F) and Cellular Component (C). In total 5682 GO terms were distributed between level 2 and 12: 20002 sequences were involved in biological processes, with a peak at level 6, 21054 and 19508 transcripts had a biological function and cellular component respectively with a peak at level 7. (TIF) [file pone.0178119.s009.tif]

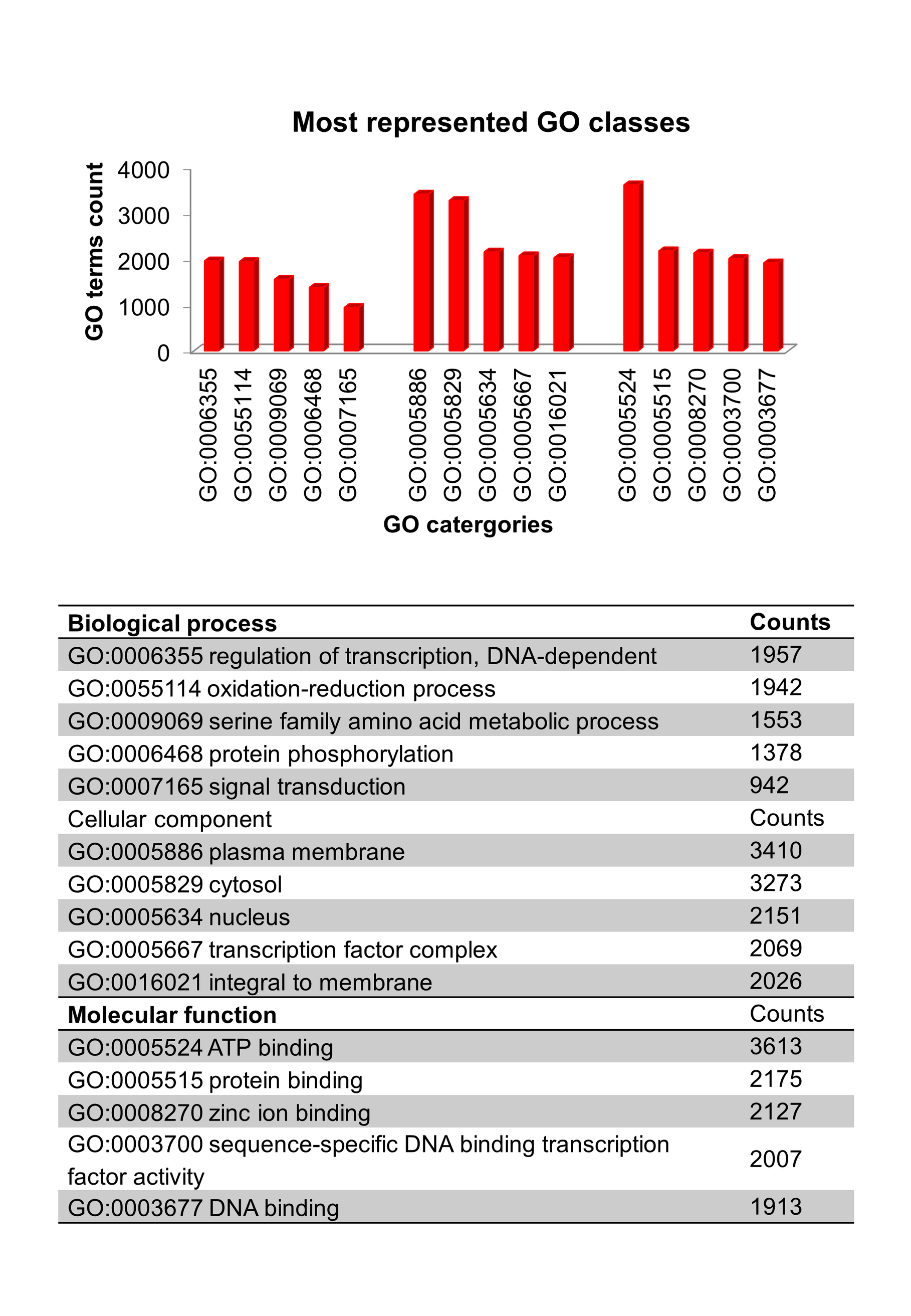

Supplement: S3 Fig — From left to the right, the 5 GO terms with the highest counts in biological process, cellular component and molecular function are shown. (TIF) [file pone.0178119.s010.tif]

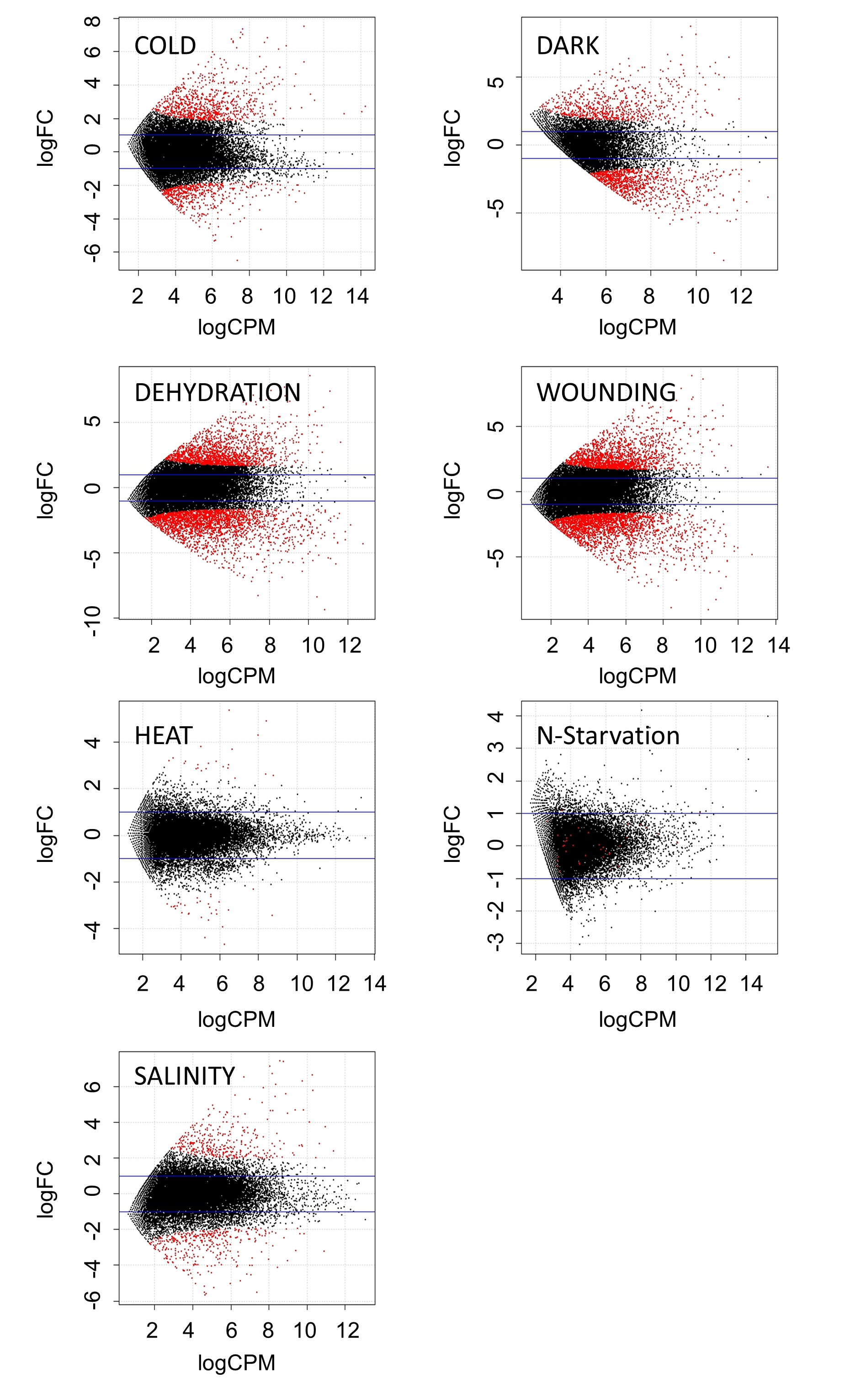

Supplement: S4 Fig — Each plot shows the gene expression as logFC ratio versus abundance in CPM of each transcript for each treatment versus the control. Each dot represents a gene. Points representing significantly (FDR ≤ 0.05) differentially down-regulated and up-regulated genes are shown in red below -2 FC or above 2 FC respectively. Blue lines indicate a logFC = 1. (TIF) [file pone.0178119.s011.tif]

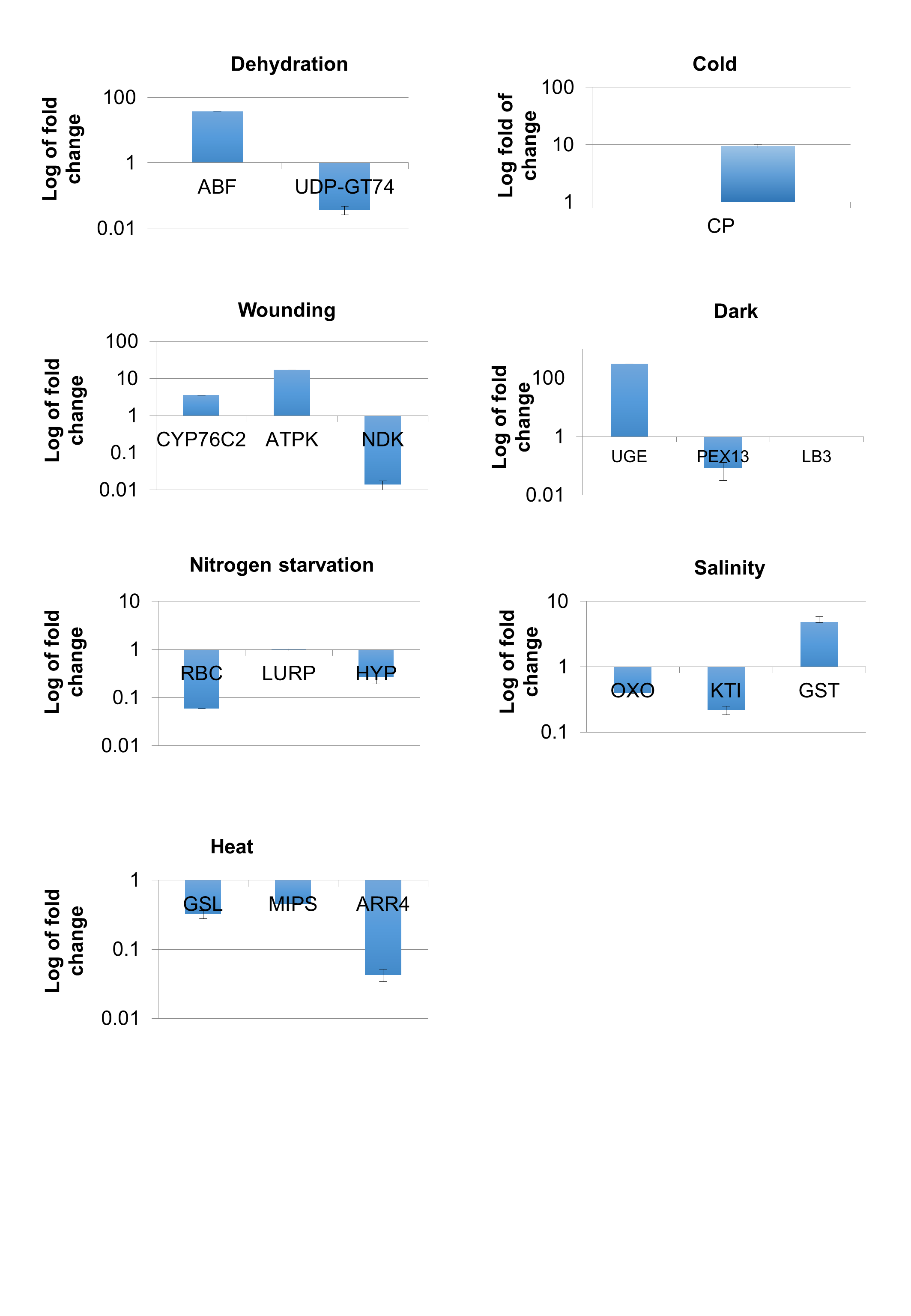

Supplement: S5 Fig — 18 genes were selected across the seven stressed libraries and real time RT-PCR performed as described in the methods section (mean ± S.E.; n = 3). (TIF) [file pone.0178119.s012.tif]

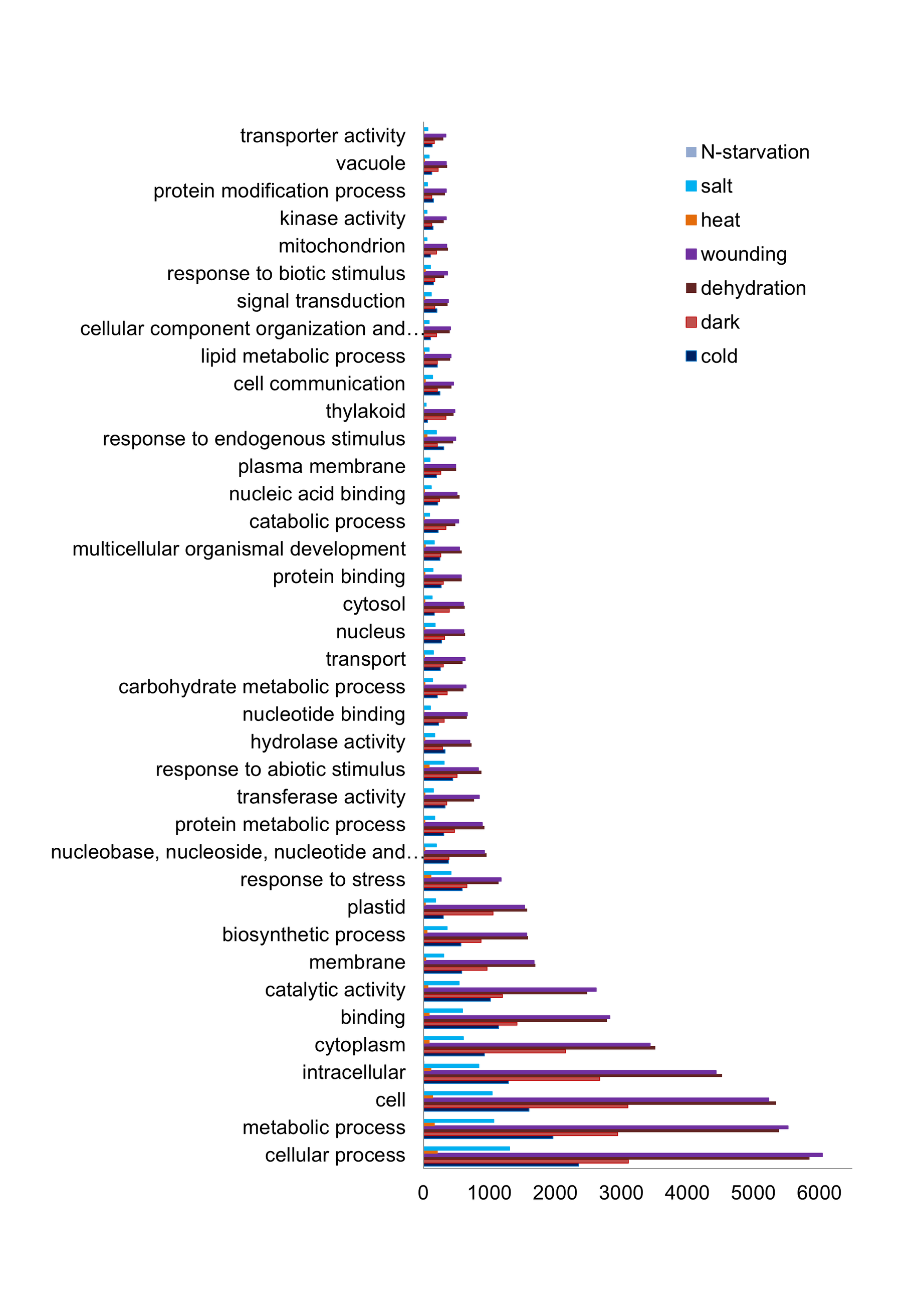

Supplement: S6 Fig — The chart shows the most enriched GO slim terms for each stress. (TIF) [file pone.0178119.s013.tif]

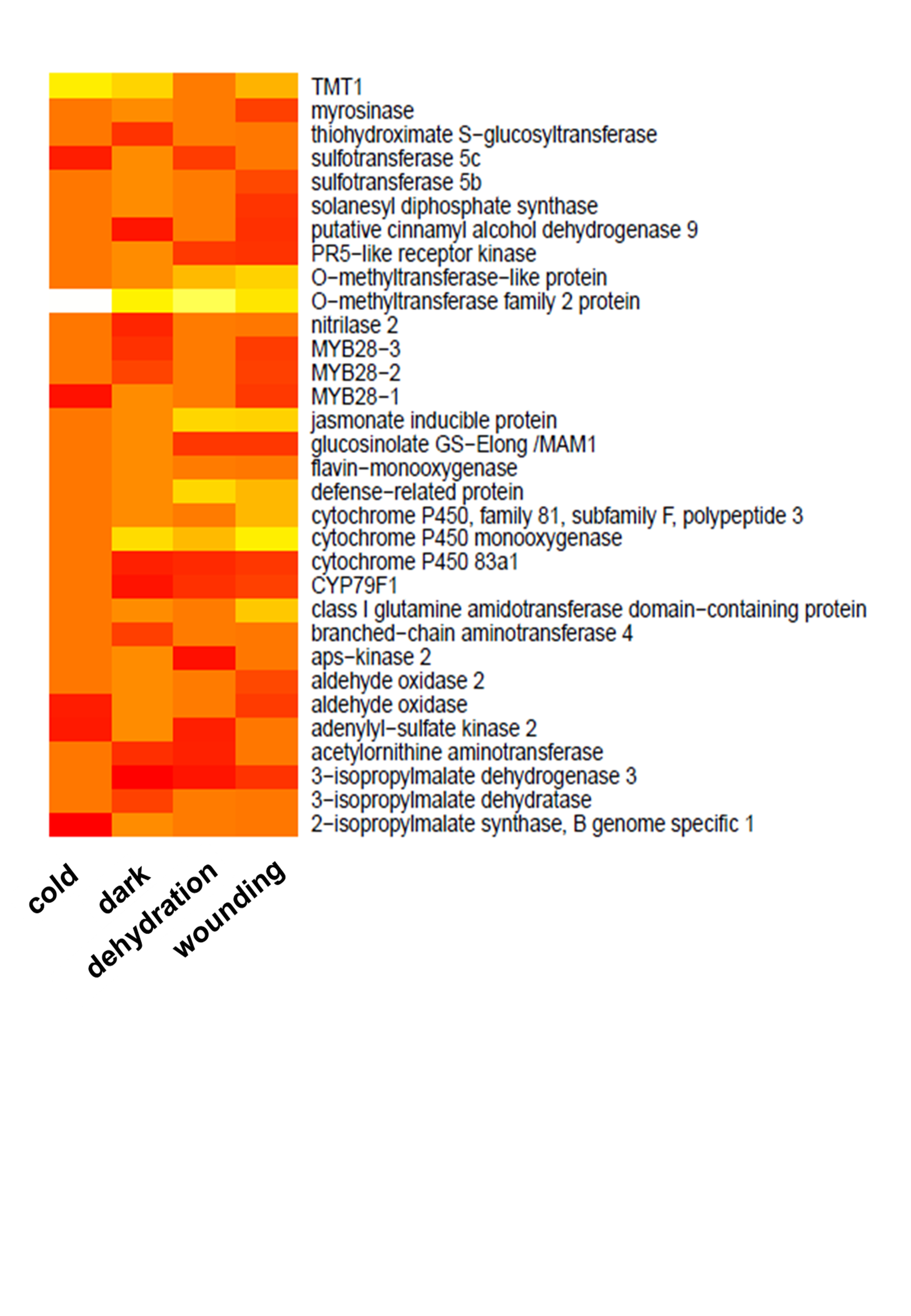

Supplement: S7 Fig — Heatmap of the log FC calculated with edgeR. The bar colour reflects the FC levels. Dark and light shades indicate lower FC and higher FC respectively. (TIF) [file pone.0178119.s014.tif]
